# Efficient Unitary Designs with a System-Size Independent Number of Non-Clifford Gates

**DOI:** 10.1007/s00220-022-04507-6

**Published:** 2022-11-12

**Authors:** J. Haferkamp, F. Montealegre-Mora, M. Heinrich, J. Eisert, D. Gross, I. Roth

**Affiliations:** 1 Dahlem Center for Complex Quantum Systems, Freie Universität Berlin, Berlin, Germany; 2 Institute for Theoretical Physics, University of Cologne, Cologne, Germany; 3 Quantum Technology Research Group, Heinrich Heine University Düsseldorf, Düsseldorf, Germany; 4 Quantum Research Centre, Technology Innovation Institute, Abu Dhabi, UAE

## Abstract

Many quantum information protocols require the implementation of random unitaries. Because it takes exponential resources to produce Haar-random unitaries drawn from the full *n*-qubit group, one often resorts to *t*-designs. Unitary *t*-designs mimic the Haar-measure up to *t*-th moments. It is known that Clifford operations can implement at most 3-designs. In this work, we quantify the non-Clifford resources required to break this barrier. We find that it suffices to inject $O(t^{4}\log ^{2}(t)\log (1/\varepsilon ))$ many non-Clifford gates into a polynomial-depth random Clifford circuit to obtain an $\varepsilon$-approximate *t*-design. Strikingly, the number of non-Clifford gates required is independent of the system size – asymptotically, the density of non-Clifford gates is allowed to tend to zero. We also derive novel bounds on the convergence time of random Clifford circuits to the *t*-th moment of the uniform distribution on the Clifford group. Our proofs exploit a recently developed variant of Schur-Weyl duality for the Clifford group, as well as bounds on restricted spectral gaps of averaging operators.

Random vectors and unitaries are ubiquitous in protocols and arguments of quantum information and many-body physics. In quantum information, a paradigmatic example is the *randomized benchmarking protocol* [1–3], which aims to characterize the error rate of quantum gates. There, random unitaries are used to average potentially complex errors into a single, easy to measure error rate. In many-body physics, random unitaries are used e.g. to model the dynamics that are thought to describe the mixing process that quantum information undergoes when absorbed into, and evaporated from, a black hole [4]. In these and related cases, one is faced with the issue that unitaries drawn uniformly from the full many-body group are *unphysical* in the sense that, with overwhelming probability, they cannot be implemented efficiently. The notion of a *unitary* *t*-*design* captures an efficiently realizable version of uniform randomness [5–7]. More specifically, a probability measure on the unitary group is a *t*-design if it matches the uniform Haar measure up to *t*-th moments.

Applications abound. The randomness provided by designs is used to foil attackers in quantum cryptography protocols [8–10]. It guards against worst case behavior in various quantum [10–16] and classical [17] estimation problems. Designs allow for an efficient implementation of *decoupling* procedures, a primitive in quantum Shannon theory [18]. In quantum complexity, unitary designs are used as models for generic instances of time evolution that display a quantum computational speed-up [19, 20]. Unitary designs are now standard tools for the quantitative study of toy models in high energy physics, quantum gravity, and quantum thermodynamics [4, 21–23].

The multitude of applications motivates the search for efficient constructions of unitary *t*-designs [24–28]. In particular, Brandao, Harrow and Horodecki [24] show that local random circuits on *n* qubits with $O(n^2t^{10})$ many gates give rise to an approximate *t*-design. In practice, it is often desirable to find more structured implementations. Designs consisting of *Clifford operations* would be particular attractive from various points of view: (i) Because the Clifford unitaries form a finite group, elements can be represented exactly using a small number ($O(n^2)$) of bits. (ii) The Gottesman-Knill Theorem ensures that there are efficient classical algorithms for simulating Clifford circuits. (iii) Most importantly, in *fault-tolerant architectures* [29, 30], Clifford unitaries tend to have comparatively simple realizations, while the robust implementation of general gates (e.g. via *magic-state distillation*) carries a significant overhead. The difference is so stark that in this context, Clifford operations are often considered to be a free resource, and the complexity of a circuit is measured solely in terms of the number of non-Clifford gates [31, 32].

The Clifford group is known to form a unitary *t*-design for $t=2$ [9] and $t=3$ [33–35], but fails to have this property for $t>3$ [33–37]. In fact, the Clifford group is singled out among the finite subgroups of the unitary group by being a 3-design [38]. Moreover, Refs. [38, 39] together imply that *any* local gate set that generates an exact unitary design of order $t>3$ must necessarily be universal, c.f. the discussion in Sect. 5. Hence, any efficient design construction for $t>3$ can only be approximate, and the Clifford group seems to be a distinguished starting point.

This leads us to the central question underlying this work: *How many non-Clifford gates are required to generate an approximate unitary* *t*-*design?* A direct application of the random circuit model of Ref. [24] yields an estimate of $O(n^2t^{10})$ non-Clifford operations. In this paper we show that a polynomial-sized random Clifford circuit, together with a *system size-independent* number of $O(t^{4}\log ^2(t))$ non-Clifford gates – a “homeopathic dose” – is already sufficient.

**Fig. 1:**
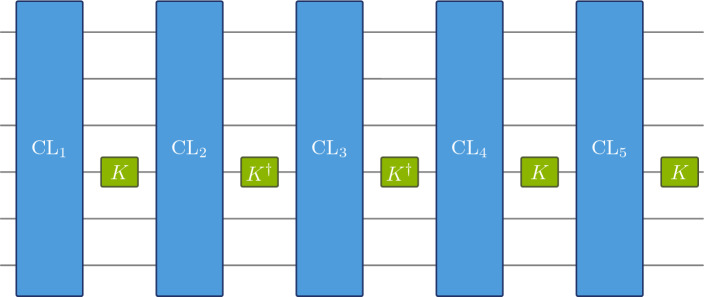
*K*-interleaved Clifford circuits: We consider a model where random Clifford operations are alternated with a non-Clifford gate *K* or its inverse $K^\dagger$

We establish this main result for two different circuit models (Fig. 1). In Sect. 1.1, we consider alternating unitaries drawn uniformly from the Clifford group with a non-Clifford gate. This gives rise to an efficient quantum circuit, as there are classical algorithms for sampling uniformly from the Clifford group, and for producing an efficient gate decomposition of the resulting operation [40]. A somewhat simpler model is analyzed in Sect. 1.2. There, we assume that the Clifford layers are circuits consisting of gates drawn form a local Clifford gate set. These circuits will only approximate the uniform measure on the Clifford group. Theorem 2, which might be of independent interest, gives novel bounds on the convergence rate.

The key to this scaling lies in the structure of the commutant of the *t*-th tensor power of the Clifford group, described by a variant of Schur-Weyl duality developed in a sequence of recent works [36, 41–43]. There, it has been shown that the dimension of this commutant – which measures the failure of the Clifford group to be a *t*-design from a representation theoretical perspective – is independent of the system size. Refs. [36, 42] have used this insight to provide a construction for exact *spherical* *t*-designs that consist of a system size-independent number of Clifford orbits. It has been left as an open problem whether these ideas can be generalized from spherical designs to the more complex notion of unitary designs, and whether the construction can be made efficient [42]. The present work resolves this question in the affirmative.

Finally, we note that in Ref. [44], it has been observed numerically that adding a single *T* gate to a random Clifford circuit has dramatic effects on the entanglement spectrum. A relation to *t*-designs was suspected. Our result provides a rigorous understanding of this observation.

## Results

### Approximate *t*-designs with few non-Clifford gates

To state our results precisely, we need to formalize the relevant notion of approximation, as well as the circuit model used. Let $\nu$ be a probability measure on the unitary group *U*(*d*). The measure $\nu$ gives rise to a quantum channel

1 $$\begin{aligned} \textrm{M}_t(\nu )(\rho ) :=\int _{{{\,\textrm{U}\,}}(d)}U^{\otimes t}\rho \left( U^{\dagger }\right) ^{\otimes t}\textrm{d}\nu (U), \end{aligned}$$

which applies $U^{\otimes t}$, with *U* chosen according to $\nu$. We will refer to $\textrm{M}_t(\nu )$ as the *t*-*th moment operator* associated with $\nu$. Following Ref. [27], we quantify the degree to which a measure approximates a *t*-design by the diamond norm distance of its moment operator to the moment operator of the Haar measure $\mu _{\textrm{H}}$ on *U*(*d*).

#### Definition 1

(*Approximate unitary design*). Let $\nu$ be a distribution on ${{\,\textrm{U}\,}}(d)$. Then $\nu$ is an (additive) $\varepsilon$-approximate *t*-design if

2 $$\begin{aligned} \Vert \textrm{M}_t(\nu )-\textrm{M}_t(\mu _\textrm{H})\Vert _{\diamond }\le \varepsilon . \end{aligned}$$

Denote the uniform measure on the multiqubit Clifford group $\textrm{Cl}(2^n)$ by $\mu _{\textrm{Cl}}$, and let *K* be some fixed single-qubit non-Clifford gate. The circuit model we are considering (Fig. 1) interleaves Clifford unitaries drawn from $\mu _{\textrm{Cl}}$, with random gates from $\{K, K^\dagger ,\mathbbm {1}\}$ acting on an arbitrary qubit.^1^ Note that the concatenation of two unitaries drawn from measures $\nu _1$ and $\nu _2$ is described by the convolution $\nu _1*\nu _2$ of the respective measures. We thus arrive at this formal definition of the circuit model:

#### Definition 2

(*K*-*interleaved Clifford circuits*). Let $K\in U(2)$. Consider the probability measure $\xi _K$ that draws uniformly from the set $\{K\otimes \mathbbm {1}_{2^{n-1}}, K^{\dagger }\otimes \mathbbm {1}_{2^{n-1}}, \mathbbm {1}_{2^n}\}$. A *K*-*interleaved Clifford circuit of depth* *k* is the random circuit acting on *n* qubits described by the probability distribution

3 $$\begin{aligned} \sigma _{k}:= \underbrace{\mu _{\textrm{Cl}}*\xi _K*\dots *\mu _\textrm{Cl}*\xi _K}_{k\; \text {times}}. \end{aligned}$$

For convenience, we work with the logarithm of base 2: $\log (x):=\log _2(x)$. We are now equipped to state the main result of this work in the form of a theorem:

#### Theorem 1

(Unitary designs with few non-Clifford gates). Let $K \in U(2)$ be a non-Clifford unitary. There are constants $C_1(K), C_2(K)$ such that for any $k\ge C_1(K)\log ^2(t)(t^{4}+t\log (1/\varepsilon ))$, a *K*-interleaved Clifford circuit with depth *k* acting on *n* qubits is an additive $\varepsilon$-approximate *t*-design for all $n\ge C_2(K)t^2$.

We give the proofs of this theorem in Sect. 3. In Theorem 1, we consider uniformly drawn multiqubit Clifford unitaries. This can be achieved with $O(n^3)$ classical random bits [40] and then implemented with $O(n^2/\log (n))$ gates [45]. Combined with these results, Theorem 1 implies an overall gate count of $O(n^2/\log (n)t^4\log ^2(t))$ improving the scaling compared to Ref. [24] in the dependence on both *t* and *n*. In this sense, our construction can be seen as a classical-quantum hybrid construction of unitary designs: The scaling is significantly improved by outsourcing as many tasks as possible to a classical computer. A construction in which all parts of the random unitary are local random circuits is considered in Corollary 2.

For designs generated from general random local circuits, numerical results suggest that convergence is much faster in practice than indicated by the proven bounds [46]. We expect that a similar effect occurs here, and that in fact very shallow *K*-interleaved Clifford circuits are sufficient to approximate *t*-designs. This intuition is supported by the numerical results of Ref. [44], which show that even a single *T*-gate has dramatic effects on the entanglement spectrum of a quantum circuit.

It is moreover noteworthy that circuits with few *T*-gates can be efficiently simulated [47–51]. The scaling of these algorithms is polynomial in the depth of the circuit, but exponential in the number of *T*-gates. Combined with our result, this implies that for fixed additive errors $\varepsilon$, there are families of $\varepsilon$-approximate unitary $O(\log (n))$-designs simulable in quasi-polynomial time. For the general random quantum circuit model, it is conjectured that a depth of order *O*(*nt*) suffices to approximate *t*-designs [24, 52]. If such a linear scaling is sufficient in our model, the quasi-polynomial time estimate for classical simulations would improve to polynomial.

For the proof of Theorem 1 we need to analyse the connection between the *t*-th moment operator of the Haar measure and the commutant of the diagonal action of the Clifford group. The latter was proven to be spanned by representations of so-called *stochastic Lagrangian sub-spaces* in Ref. [42]. In particular, we prove almost tight bounds on the overlap of the Haar operator with these basis vectors in Lemma 13 that might be of independent interest. This will allow us to invoke a powerful theorem by Varjú [53] on restricted spectral gaps of probability distributions on compact Lie groups to show that non-Clifford unitaries have a strong impact on representations of Lagrangian sub-spaces that are not also permutations. We combine this insight with a careful combinatorial argument about the Gram-Schmidt orthogonalization of the basis corresponding to stochastic Lagrangian sub-spaces to bound the difference to a unitary *t*-design in diamond norm.

Moreover, the bound for Theorem 1 allows us to prove a corollary about the stronger notion of *relative approximate designs*:

#### Definition 3

(*Relative* $\varepsilon$-*approximate* *t*-*design*). We call a probability $\nu$ a relative $\varepsilon$-approximate *t*-design if

4 $$\begin{aligned} (1-\varepsilon )\textrm{M}_t(\nu )\preccurlyeq \textrm{M}_t(\mu _{\textrm{H}})\preccurlyeq (1+\varepsilon )\textrm{M}_t(\nu ), \end{aligned}$$

where $A\preccurlyeq B$ if and only if $B-A$ is completely positive.

#### Corollary 1

(*K*-interleaved Clifford circuits as relative $\varepsilon$-approximate *t*-designs). There are constants $C_1'(K), C_2'(K)$ such that a *K*-interleaved Clifford circuit is a relative $\varepsilon$-approximate *t*-design in depth $k\ge C_1'(K)\log ^{2}(t)(2nt+\log (1/\varepsilon ))$ for all $n\ge C_2'(K)t^2$.

Hence, if we drop the system-size independence, we can achieve a scaling of *O*(*nt*) at least until $t~\sim \sqrt{n}$.

While we believe the setting of *K*-interleaved Clifford circuits to be the more relevant case, the same method of proof works for *Haar*-interleaved Clifford circuits. Here, we draw not from the gate set $\{K_i,K^{\dagger }_i,\mathbbm {1}\}$, but instead Haar-randomly from *U*(2). The advantage is that we obtain explicit constants for the depth, while the depth in the *K*-interleaved setting has to depend on a constant (as *K* might be arbitrarily close to the identity).

#### Proposition 1

(Haar-interleaved Clifford circuits as additive $\varepsilon$-approximate *t*-designs). For $k\ge 36(33t^4+3t\log (1/\varepsilon ))$, Haar-interleaved Clifford circuits with depth *k* form an additive $\varepsilon$-approximate *t*-design for all $n\ge 32t^2+7$.

Similarly, variants of Corollary 1 for Haar-interleaved Clifford circuits can be obtained, here also without the $\log ^{2}(t)$ dependence. Finally, we discuss an application to higher Rényi entropies in “Appendix D”.

### Local random Clifford circuits for Clifford and unitary designs

The circuits considered in the previous section require one to find the gate decomposition of a random Clifford operation. In this section, we analyze the case where the Clifford layers are circuits consisting of gates drawn from a local set of generators.

As a first step, we establish that a 2-local random Clifford circuit on *n* qubits of depth $O(n^2t^{9}\log ^{-2}(t)\log (1/\varepsilon ))$ constitutes a relative $\varepsilon$-approximate Clifford *t*-design, i.e., reproduces the moment operator of the Clifford group up to the *t*-th order with a relative error of $\varepsilon$. We consider local random Clifford circuits that consist of 2-local quantum gates from a finite set *G* with is closed under taking the inverse and generates ${{\,\textrm{Cl}\,}}(4)$. We refer to such a set as a *closed, generating set*. A canonical example for such a closed, generating set is $\{H \otimes \mathbbm {1}, S \otimes \mathbbm {1}, S^3 \otimes \mathbbm {1},\textrm{CX} \}$ where *H* is the Hadamard gate, *S* is the phase gate and $\textrm{CX}$ is the cNOT-gate [54]. Such a set *G* induces a set of multi-qubit Clifford unitaries ${\hat{G}} \subset {{\,\textrm{Cl}\,}}(n)$ by acting on any pair of adjacent qubits on a line, where we adopt periodic boundary conditions. We then define the corresponding random Clifford circuits.

#### Definition 4

(*Local random Clifford circuit*). Let $G \subset {{\,\textrm{Cl}\,}}(4)$ be a closed, generating set containing the identity. Define the probability measure $\sigma _G$ as the measure having uniform support on ${\hat{G}} \subset {{\,\textrm{Cl}\,}}(n)$ acting on *n* qubits. A *local random Clifford circuit* of depth *m* is the random circuits described by the probability measure $\sigma _G^{*m}$.

For technical reasons, we again assume that the identity is part of the generating set. This assumption can be avoided but simplifies the argumentation in the following. As for the Definition 2 of *K*-interleaved Clifford circuits before, any upper bound on the depth of local random Clifford circuits with identity is a bound for those without.

Our result on local random Clifford circuits even holds for a stronger notion for approximations of designs, namely relative approximate designs. Write $A\preccurlyeq B$ if $B-A$ is positive semi-definite.

#### Definition 5

(*Relative approximate Clifford t-designs*). Let $\nu$ be a probability measure on $\textrm{Cl}(2^n)$. Then, $\nu$ is a *relative* $\varepsilon$-*approximate Clifford* *t*-*design* if

5 $$\begin{aligned} (1-\varepsilon )\textrm{M}_t(\mu _{\textrm{Cl}})\preccurlyeq \textrm{M}_t(\nu ) \preccurlyeq (1+\varepsilon )\textrm{M}_t(\mu _{\textrm{Cl}}). \end{aligned}$$

With this definition, our result reads as follows.

#### Theorem 2

(Local random Clifford designs). Let $n\ge 12t$, then a local random Clifford circuit of depth $O(n\log ^{-2}(t)t^{8}(2nt+\log (1/\varepsilon )))$ constitutes a relative $\varepsilon$-approximate Clifford *t*-design.

The proof of the theorem is given in Sect. 4. This result is a significant improvement over the scaling of $O(n^8)$, which is implicit in Ref. [9].

We can combine this result with the bounds obtained in Sect. 3. To this end, consider a random circuit that *k*-times alternatingly applies a local random Clifford circuit of depth *m*, and a unitary drawn from the probability measure $\xi _K$. The corresponding probability measure is

6 $$\begin{aligned} \sigma _{k,m}:= \underbrace{\sigma _G^{*m}*\xi _K*\dots *\sigma _G^{*m}*\xi _K}_{k\;\text {times}}. \end{aligned}$$

For these local random circuits we establish the following result:

#### Corollary 2

(Local random unitary design). Let $K \in U(2)$ be a non-Clifford gate and let $G \subset {{\,\textrm{Cl}\,}}(4)$ be a closed, generating set. There are constants $C''_1(K,G), C''_2(K), C''_3(K)$ such that whenever

$$\begin{aligned} m \ge C''_1(K,G)n \log ^{-2}(t) t^8\left( 2nt + \log (1/\varepsilon ) \right) \ \text {and}\ k\ge C''_2(K)\log ^2(t)(t^{4}+t\log (1/\varepsilon )), \end{aligned}$$

the local random circuit $\sigma _{k,m}$, defined in (6), is an $\varepsilon$-approximate unitary *t*-design for all $n\ge C''_3(K)t^2$.

The complete argument for the corollary is given at the end of Sect. 4. After introducing technical preliminaries in Sect. 2, the remainder of the paper, Sect. 3 and Sect. 4, is devoted to the proofs of Theorem 1, Theorem 2 and the Corollary 2. Finally, in Sect. 5 we elaborate on and formalize as Proposition 3 the observation that there exists no non-universal gate set generating exact 4-designs for arbitrary system size. This observation is an immediate consequence of the classification of finite unitary *t*-groups and a criterion for the universality of finite gate sets [38, 39, 55].

## Technical Preliminaries

### Operators and superoperators

Given a (finite-dimensional) Hilbert space ${\mathcal {H}}$, we denote with $L({\mathcal {H}})$ the space of linear operators on ${\mathcal {H}}$ with involution $\dagger$ mapping an operator to its adjoint with respect to the inner product on ${\mathcal {H}}$. $L({\mathcal {H}})$ naturally inherits a Hermitian inner product, the *Hilbert-Schmidt inner product*

7 $$\begin{aligned} \left( A \big | B \right) := {{\,\textrm{Tr}\,}}(A^\dagger B), \qquad \forall A,B\in L(\mathcal H). \end{aligned}$$

As this definition already suggests, we will use “operator kets and bras” whenever we think it simplifies the notation. Concretely, we write $\left. \left| {B}\right. \right) =B$ and denote with $\left. \left( {A}\right. \right|$ the linear form on $L({\mathcal {H}})$ given by

8 $$\begin{aligned} \left. \left( {A}\right. \right| :\; B \longmapsto \left( A \big | B \right) . \end{aligned}$$

Following common terminology in quantum information theory, we call linear maps $\phi :\,L({\mathcal {H}})\rightarrow L({\mathcal {H}})$ on operators “superoperators”. We use $\phi ^\dagger$ to denote the adjoint map with respect to the Hilbert-Schmidt inner product. Note that with the above notation, $\phi = \left. \left| {A}\right. \right) \!\! \left. \left( {B}\right. \right|$ defines a rank one superoperator with $\phi ^\dagger = \left. \left| {B}\right. \right) \!\! \left. \left( {A}\right. \right|$. Moreover, we will denote by the superoperator ${{\,\textrm{Ad}\,}}_A := A\cdot A^{-1}$ the *adjoint action* of an invertible operator $A\in {{\,\textrm{GL}\,}}(\mathcal H)$ on $L({\mathcal {H}})$. For notational reasons, we sometimes write ${{\,\textrm{Ad}\,}}(A)$ instead of ${{\,\textrm{Ad}\,}}_A$.

We consistently reserve the notation $\left\| \cdot \right\| _p$ for the Schatten *p*-norms

9 $$\begin{aligned} \left\| A \right\| _p := {{\,\textrm{Tr}\,}}(|A|^p)^{1/p} = \left\| \sigma (A) \right\| _{\ell _p}, \end{aligned}$$

where $\sigma (A)$ is the vector of singular values of *A*. In particular, we use the *trace norm* $p=1$, the *Frobenius* or *Hilbert-Schmidt norm* $p=2$ and the *spectral norm* $p=\infty$. Clearly, this norms can be defined for both operators and superoperators and we will use the same symbol in both cases. For the latter, however, there is also a family of induced operator norms

10 $$\begin{aligned} \left\| \phi \right\| _{p\rightarrow q} := \sup _{\left\| X \right\| _p \le 1} \left\| \phi (X) \right\| _q. \end{aligned}$$

Note that $\left\| \cdot \right\| _{2\rightarrow 2} \equiv \left\| \cdot \right\| _{\infty }$. Finally, we are interested in “stabilized” versions of these induced norms, in particular the *diamond norm*

11 $$\begin{aligned} \left\| \phi \right\| _\diamond&:=\sup _{d\in {\mathbb {N}}} \left\| \phi \otimes \textrm{id}_{L({\mathbb {C}}^d)} \right\| _{1\rightarrow 1} = \left\| \phi \otimes \textrm{id}_{L({\mathcal {H}})} \right\| _{1\rightarrow 1}. \end{aligned}$$

The following norm inequality will be useful [56]

12 $$\begin{aligned} \left\| \phi \right\| _\diamond \le (\dim {\mathcal {H}})^2 \left\| \phi \right\| _\infty , \qquad \left\| \phi \right\| _\infty \le \sqrt{\dim {\mathcal {H}}} \left\| \phi \right\| _\diamond . \end{aligned}$$

### Commutant of the diagonal representation of the Clifford group

In this section, we review some of the machinery developed in Ref. [42]. Recall that the *n*-qubit *Clifford group* ${{\,\textrm{Cl}\,}}(n)$ is defined as the unitary normalizer of the Pauli group ${\mathcal {P}}_n$ as

13 $$\begin{aligned} {{\,\textrm{Cl}\,}}(n) = \left\{ U \in U(2^n, {\mathbb {Q}}[i]) \; \big | \; U{\mathcal {P}}_n U^\dagger \subset {\mathcal {P}}_n \right\} . \end{aligned}$$

Here, we followed the convention to restrict the matrix entries to rational complex numbers. This avoids the unnecessary complications from an infinite center *U*(1) yielding a finite group with minimal center $Z({{\,\textrm{Cl}\,}}(n))=Z({\mathcal {P}}_n)\simeq {\mathbb {Z}}_4$. The Clifford group can equivalently be defined in a less conceptual but more constructive manner: It is the subgroup of ${{\,\textrm{U}\,}}(2^n)$ generated by $\textrm{CX}$, the controlled not gate, the Hadamard gate *H* and the phase gate *S*.

For this work, the *t*-th diagonal representation of the Clifford group, defined as

14 $$\begin{aligned} \tau ^{(t)}:\; {{\,\textrm{Cl}\,}}(n) \longrightarrow {{\,\textrm{U}\,}}(2^{nt}), \quad U \longmapsto U^{\otimes t}, \end{aligned}$$

will be of major importance. It acts naturally on the Hilbert space $(({\mathbb {C}}^2)^{\otimes n})^{\otimes t}$ which can be seen as *t* copies of an *n*-qubit system. However, it will turn out that the operators commuting with this representation naturally factorize with respect to a different tensor structure on this Hilbert space, namely $(({\mathbb {C}}^2)^{\otimes t})^{\otimes n}\simeq (({\mathbb {C}}^2)^{\otimes n})^{\otimes t}$. Because of the different exponents, it should be clear from the context which tensor structure is meant. We will make ubiquitous use of the description of the commutant of the diagonal representation in terms of *stochastic Lagrangian sub-spaces* [42]:

#### Definition 6

(*Stochastic Lagrangian sub-spaces*). Consider the quadratic form ${\mathfrak {q}}:{\mathbb {Z}}^{2t}_2\rightarrow {\mathbb {Z}}_{4}$ defined as ${\mathfrak {q}}(x,y):= x\cdot x-y\cdot y \mod 4$. The set $\Sigma _{t,t}$ denotes the set of all sub-spaces $T\subseteq {\mathbb {Z}}^{2t}_2$ being subject to the following properties:

1. T is totally ${\mathfrak {q}}$-isotropic: $x\cdot x=y\cdot y \mod 4$ for all $(x,y)\in T$.
2. T has dimension *t* (the maximum dimension compatible with total isotropicity).
3. T is *stochastic*: $$(1,\dots , 1)\in T$$.

We call elements in $\Sigma _{t,t}$ *stochastic Lagrangian sub-spaces*. We have

15 $$\begin{aligned} |\Sigma _{t,t}|=\prod _{k=0}^{t-2}(2^k+1)\le 2^{\frac{1}{2} (t^2+5t)}. \end{aligned}$$

With this notion, we can now state the following key theorem from Ref. [42].

#### Theorem 3

([42]). If $n\ge t-1$, then the commutant $\tau ^{(t)}({{\,\textrm{Cl}\,}}(n))'$ of the *t*-th diagonal representation of the Clifford group is spanned by the linearly independent operators $r(T)^{\otimes n}$, where $T\in \Sigma _{t,t}$ and

16 $$\begin{aligned} r(T):= \sum _{(x,y)\in T} |x\rangle \langle y|. \end{aligned}$$

Since the representation in question is fixed throughout this paper, we will simplify the notation from now on and write ${{\,\textrm{Cl}\,}}(n)'\equiv \tau ^{(t)}({{\,\textrm{Cl}\,}}(n))'$. To make use of a more sophisticated characterization of the elements *r*(*T*) developed in Ref. [42, Section 4], we need the following definitions.

#### Definition 7

(*Stochastic orthogonal group*). Consider the quadratic form $q:{\mathbb {Z}}^t_2\rightarrow {\mathbb {Z}}_4$ defined as $q(x):=x\cdot x \mod 4$. The *stochastic orthogonal group* $O_t$ is defined as the group of $t\times t$ matrices *O* with entries in ${\mathbb {Z}}_2$ such that $q(Ox)=q(x)$ for all $x\in {\mathbb {Z}}^t_2$.

The subspace $T_O:=\{(Ox,x),x\in {\mathbb {Z}}_2^t\}$ is a stochastic Lagrangian subspace. Moreover, the operator $r(O):=r(T_O)$ is unitary. We will therefore canonically embed the orthogonal stochastic group $O_t\subset \Sigma _{t,t}$. Notice that the permutation group on *t* objects, referred to as $S_t$, may be embedded into $O_t$ by acting on the standard basis of ${\mathbb {Z}}_2^t$. Together with $O_t$, the following definition can be used to fully characterize the set of stochastic Langrangian sub-spaces, $\Sigma _{t,t}$.

#### Definition 8

(*Defect sub-spaces*). A defect subspace is a subspace $N\subseteq {\mathbb {Z}}_2^t$ which is isotropic with respect to *q*, that is, that $q(x)=0$ for all $x\in N$.

The quadratic form *q* is what is known as a *generalized quadratic refinement* of the bi-linear form defined by the inner product $(x,y)\mapsto x\cdot y \mod 2$ (see, e.g., Ref. [57, App. A] for a self-contained discussion). In the following, the ortho-complement $N^\perp$ of a subspace $N\subseteq {\mathbb {Z}}_2^t$ is taken with respect to the inner product modulo 2,

$$\begin{aligned} N^\perp = \{v\in {\mathbb {Z}}_2^t \;|\; v\cdot u = 0\mod 2,\ \forall \ u\in N\}. \end{aligned}$$

Notice that $q(x)=0$ implies that $x\cdot {\textbf{1}}_t=0\mod 2$, where ${\textbf{1}}_t:=(1,\dots ,1)^T$ is the all-ones vector. Thus, we do not need a separate clause requiring ${\textbf{1}}_t\in N^\perp$ in the definition of defect sub-spaces (compare Ref. [42, Def. 4.16]). Moreover, one may verify that $2q(x)=2x\cdot {\textbf{1}}_t\mod 4$. This implies, similarly, that if *O* preserves *q*, then $O{\textbf{1}}_t={\textbf{1}}_t$. Borrowing the language of [42], all *q*-isometries are stochastic (compare the definition of the orthogonal stochastic group in that reference, [42, Def. 4.11]). The reason for these simplifications is that here we focus on the qubit case exclusively, while Ref. [42] works simultaneously for qubits and odd qudits. We use the names *stochastic orthogonal group* and *defect subspace* (rather than simply *q*-*isometry group* and *isotropic subspace*) to keep with the notation of that reference.

For any defect subspace *N*, it holds that $N\subseteq N^{\perp }$ (and thus $\dim N\le t/2$). Because of this, defect sub-spaces $N\subseteq {\mathbb {Z}}_2^t$ define *Calderbank-Shor-Sloane (CSS)* codes

17 $$\begin{aligned} \textrm{CSS}(N):=\left\{ Z(p)X(q) \; | \; q,p\in N\right\} , \end{aligned}$$

where the action of the multi-qubit Pauli operators is $Z(p)\left. \left| {x}\right. \right\rangle :=(-1)^{p\cdot x}\left. \left| {x}\right. \right\rangle$ and $X(q)\left. \left| {x}\right. \right\rangle :=\left. \left| {x+q}\right. \right\rangle$ for $x\in {\mathbb {Z}}_2^t$. The corresponding projector is given by

18 $$\begin{aligned} P_N:=P_{\textrm{CSS}(N)}=\frac{1}{|N|^2}\sum _{q,p\in N}Z(p)X(q). \end{aligned}$$

Since the order of the stabilizer group is $2^{2\dim N}$, $P_N$ projects onto a $2^{t-2\dim N}$-dimensional subspace of $({\mathbb {C}}^2)^{\otimes t}$. For $N=\{0\}$ we set $P_{\textrm{CSS}(N)}:=\mathbbm {1}$. We summarize the findings of Ref. [42, Sect. 4] in Thm. 4. We give a short proof to give an explicit relation between this theorem and the results of that work.

#### Theorem 4

([42]). Consider $$T\in \Sigma _{t,t}$$, then

19 $$\begin{aligned} r(T)=2^{\dim N}r(O)P_{\textrm{CSS}(N)}=2^{\dim N'}P_{\textrm{CSS}(N')}r(O') \end{aligned}$$

for $O,O'\in O_t$ and $N,N'$ are unique defect sub-spaces with $\dim N=\dim N'$.

#### Proof

Recall from Ref. [42] that the code space $\textrm{range}\ P_{\textrm{CSS}(N)}$ has an orthonormal basis of coset state vectors given by

$$\begin{aligned} \left\{ \left. \left| {N,[x]}\right. \right\rangle := \frac{1}{\sqrt{N}}\sum _{y\in N} \left. \left| {x+y}\right. \right\rangle \;\Big |\; x\in N^\perp ,\ [x]\in N^\perp /N \right\} . \end{aligned}$$

One may compute that $r(O)\left. \left| {N, [x]}\right. \right\rangle = \left. \left| {ON, [Ox]}\right. \right\rangle$. This way,

$$\begin{aligned} r(O)P_{\textrm{CSS}(N)} = \sum _{[x]\in N^\perp /N} \left. \left| {ON,[Ox]}\right. \right\rangle \left. \left\langle {N,[x]}\right. \right| . \end{aligned}$$

Comparing this equation to [42, Lem. 4.23] we see that the set $\{2^{\dim N}r(O)P_{\textrm{CSS}(N)}\}_O$ is equal to the set of *r*(*T*) operators with right defect subspace given by *N*, i.e., with $T_{RD}=N$ in the notation of that reference. This way, varying over *N* we obtain the full set $\Sigma _{t,t}$. The existence of a decomposition $2^{\dim N}P_{\textrm{CSS}(N')}r(O')$ follows from the above by noting that $r(O)P_{\textrm{CSS}(N)}r(O)^\dagger = P_{\textrm{CSS}(ON)}$. $\square$

#### Lemma 1

(Norms of *r*(*T*)). Suppose $r(T)=2^{\dim N}r(O)P_{N}$ as in Theorem 4. Then it holds:

20 $$\begin{aligned} \left\| r(T) \right\| _{1}&= 2^{t-\dim N},&\left\| r(T) \right\| _{2}&= 2^{t/2},&\left\| r(T) \right\| _\infty&= 2^{\dim N}. \end{aligned}$$

#### Proof

Since any Schatten *p*-norm is unitarily invariant, we have $\left\| r(T) \right\| _p = 2^{\dim N} \left\| P_N \right\| _p$. The statements follow from ${{\,\textrm{rank}\,}}P_N = 2^{t-2\dim N}$. $\square$

In the following, we will often work with a normalized version of the *r*(*T*) operators which we define as

21 $$\begin{aligned} Q_T := \frac{r(T)}{\left\| r(T) \right\| _{2}} = 2^{-t/2} r(T). \end{aligned}$$

## Approximate Unitary *t*-Designs

In this section, we give a bound on the number of non-Clifford gates needed to leverage the Clifford group to an approximate unitary *t*-design. This is made precise by the following two theorems which rely on two distinct proof strategies and come with different trade-offs.

### Theorem 1

(Unitary designs with few non-Clifford gates). Let $K \in U(2)$ be a non-Clifford unitary. There are constants $C_1(K), C_2(K)$ such that for any $k\ge C_1(K)\log ^2(t)(t^{4}+t\log (1/\varepsilon ))$, a *K*-interleaved Clifford circuit with depth *k* acting on *n* qubits is an additive $\varepsilon$-approximate *t*-design for all $n\ge C_2(K)t^2$.

Recall from Def. 2 that a *K*-interleaved Clifford circuit has an associated probability measure $\sigma _K:=(\mu _{{{\,\textrm{Cl}\,}}} * \xi _K)^{*k}$ where $\xi _K$ is the measure which draws uniformly from $\{K,K^\dagger ,\mathbbm {1}\}$ on the first qubit. Let us introduce the notation

22 $$\begin{aligned} \textrm{R}(K):= \int _{U(2^n)} {{\,\textrm{Ad}\,}}_U^{\otimes t}\textrm{d}\xi _{k}(U)= \frac{1}{3}\left( {{\,\textrm{Ad}\,}}_K^{\otimes t}+{{\,\textrm{Ad}\,}}_{K^{\dagger }}^{\otimes t}+\textrm{id}\right) \otimes \textrm{id}_{n-1}. \end{aligned}$$

Then, our goal is to bound the deviation of the moment operator

23 $$\begin{aligned} \textrm{M}_t(\sigma _{k}) = \int _{U(2^n)} {{\,\textrm{Ad}\,}}_U^{\otimes t} \textrm{d}\sigma _{k}(U)=\underbrace{ \textrm{M}_t(\mu _\textrm{Cl})\textrm{R}(K)\dots \textrm{M}_t(\mu _{\textrm{Cl}})\textrm{R}(K)}_{k \text { times}}, \end{aligned}$$

from the Haar projector $P_\textrm{H}\equiv \textrm{M}_t(\mu _\textrm{H})$ in diamond norm. Using that $P_\textrm{H}$ is invariant under left and right multiplication with unitaries, we have the identity

24 $$\begin{aligned} A^k - P_\textrm{H}= ( A - P_\textrm{H})^k, \end{aligned}$$

for any mixed unitary channel *A*. Thus, we can rewrite the difference of moment operators as

25 $$\begin{aligned} \textrm{M}_t(\sigma _{k})-P_\textrm{H}= [P_{{{\,\textrm{Cl}\,}}}\textrm{R}(K)]^k-P_{\textrm{H}} = \left[ \left( P_{{{\,\textrm{Cl}\,}}}-P_{\textrm{H}}\right) \textrm{R}(K)\right] ^k, \end{aligned}$$

where we have introduced the shorthand notation $P_{{{\,\textrm{Cl}\,}}}:= \textrm{M}_t(\mu _{{{\,\textrm{Cl}\,}}})$.

### Remark 1

(*Non-vanishing probability of applying the identity*). We apply *K*, $K^{\dagger }$ with equal probability in Theorem 1 such that *R*(*K*) is Hermitian. The non-vanishing probability of applying $\mathbbm {1}$, i.e., of doing nothing, is necessary in the proof of Lemma 2, because we require the probability distribution $\xi _K*\xi _K$ to have non-vanishing support on a non-Clifford gate. If $\xi _K$ is the uniform measure on *K* and $K^{\dagger }$, then $\xi _K*\xi _K$ has support on $K^2$, $(K^{\dagger })^{2}$ and $\mathbbm {1}$. We can hence drop this assumption for gates that do not square to a Clifford gate. This is not the case for e.g. the *T*-gate.

Our proof strategy for Theorem 1 makes use of the following two lemmas which are proven in Sects. 6.1 and 6.2. The first lemma is key to the derivations in this section. It is based on a bound (Lemma 13) on the overlap of stochastic Lagrangian sub-spaces with the Haar projector and Theorem 5, a special case of a theorem about restricted spectral gaps of random walks on compact Lie groups due to Varjú [53].

### Lemma 2

(Overlap bound). Let *K* be a single qubit gate which is not contained in the Clifford group. Then, there is a constant $c(K)>0$ such that

26 $$\begin{aligned} \eta _{K,t}:= \max _{\begin{array}{c} T\in \Sigma _{t,t}-S_t\\ T'\in \Sigma _{t,t} \end{array}} \frac{1}{3} \left| \left( Q_T \right| {{\,\textrm{Ad}\,}}_K^{\otimes t}+{{\,\textrm{Ad}\,}}_{K^{\dagger }}^{\otimes t}+\textrm{id} \left| Q_{T'} \right) \right| \le 1-c(K)\log ^{-2}(t). \end{aligned}$$

The second lemma is of a more technical nature.

### Lemma 3

(Diamond norm bound). Consider $T_1,T_2\in \Sigma _{t,t}$ and denote with $N_1,N_2$ their respective defect spaces. Then, it holds that

27 $$\begin{aligned} \left\| \left. \left| {Q_{T_1}}\right. \right) \!\! \left. \left( {Q_{T_2}}\right. \right| \right\| _\diamond&\le 2^{\dim N_2-\dim N_1}, \end{aligned}$$

28 $$\begin{aligned} |\left( Q_{T_1} \big | Q_{T_2} \right) |&\le 2^{-|\dim N_1-\dim N_2|}. \end{aligned}$$

The difficulty of using these results to bound the difference

29 $$\begin{aligned} \textrm{M}_t(\sigma _{k})-P_\textrm{H}= \big [ \left( P_{{{\,\textrm{Cl}\,}}}-P_{\textrm{H}}\right) \textrm{R}(K)\big ]^k, \end{aligned}$$

stems from the following reason: The range of the projector $P_{{{\,\textrm{Cl}\,}}}-P_{\textrm{H}}$ is the ortho-complement of the space spanned by permutations $Q_{\pi }^{\otimes n}$ for $\pi \in S_t$ within the commutant of the Clifford group spanned by the operators $Q_T^{\otimes n}$. Although this is a conveniently factorizing and well-studied basis, it is *non-orthogonal*. Thus, the projectors do not possess a natural expansion in this basis and we can not directly use the above bounds. However, we can write it explicitly in a suitable orthonormal basis of the commutant obtained by the Gram-Schmidt procedure from the basis $\{Q_T^{\otimes n} \, | \,T \in \Sigma _{t,t}\}$. We summarize the properties of this basis in the following lemma.

### Lemma 4

(Properties of the constructed basis). Let $\{T_j\}_{j=1}^{|\Sigma _{t,t}|}$ be an enumeration of the elements of $\Sigma _{t,t}$ such that the first *t*! spaces $T_j$ correspond to the elements of $S_t$. Then, the $\{E_j\}$ constitutes an orthogonal (but not normalized) basis, where

30 $$\begin{aligned} E_j :=\sum _{i=1}^jA_{i,j}\,Q_{T_i}^{\otimes n}:= \sum _{i=1}^j\left[ \sum _{\begin{array}{c} \Pi \in S_j\\ \Pi (j)=i \end{array}}{{\,\textrm{sign}\,}}(\Pi )\prod _{l=1}^{j-1} \left( Q_{T_l} \big | Q_{T_{\Pi (l)}} \right) ^n\right] \,Q_{T_i}^{\otimes n}. \end{aligned}$$

Denote by $N_i$ the defect space of $T_i$. For $n\ge \frac{1}{2}(t^2+5t)$, we have

31 $$\begin{aligned} |A_{i,j}|&\le 2^{t^3+4t^2+6t-n|\dim N_i-\dim N_j|}, \qquad \forall i,j, \end{aligned}$$

32 $$\begin{aligned} |A_{i,j}|&\le 2^{2t^2+10t-n}, \qquad \forall i\ne j. \end{aligned}$$

Moreover, it holds that

33 $$\begin{aligned} 1-2^{t^2+7t-n}\le A_{j,j}\le 1+2^{t^2+7t-n}. \end{aligned}$$

We believe that the explicit bounds in Lemma 4 might be of independent interest in applications of the Schur-Weyl duality of the Clifford group. For the sake of readibility, and as Theorem 1 holds up to an inexplicit constant, we will bound all polynomials in *t* by their leading order term in the following. Specifically, the bounds in Lemma 4 will be simplified by using the inequalities

34 $$\begin{aligned} t^3+4t^2+6t&\le 11t^3, \end{aligned}$$

35 $$\begin{aligned} 2t^2+10t \le 12t^2&\le 12 t^3, \end{aligned}$$

36 $$\begin{aligned} t^2+7t \le 8t^2&\le 8t^3 \end{aligned}$$

which hold for all positive integers *t*.

### Proof of Theorem 1

Notice that from (25), we have the expression

37 $$\begin{aligned}&\Vert [P_{{{\,\textrm{Cl}\,}}}\textrm{R}(K)]^k-P_{\textrm{H}}\Vert _{\diamond } \end{aligned}$$

38 $$\begin{aligned}&\quad = \left\| \left[ \left( \sum _{j=t!+1}^{|\Sigma _{t,t}|}\frac{1}{\left( E_j \big | E_j \right) }\left. \left| {E_j}\right. \right) \!\! \left. \left( {E_j}\right. \right| \right) \textrm{R}(K)\right] ^k\right\| _{\diamond } \end{aligned}$$

39 $$\begin{aligned}&\quad = \left\| \sum _{j_1,\dots , j_m=t!+1}^{|\Sigma _{t,t}|}\prod _{l=1}^k \frac{1}{\left( E_{j_l} \big | E_{j_l} \right) } \left. \left| {E_{j_1}}\right. \right) \left. \left( {E_{j_1}}\right. \right| R(K)\left. \left| {E_{j_2}}\right. \right) \dots \left. \left( { E_{j_k}}\right. \right| R(K)\right\| _{\diamond } \end{aligned}$$

40 $$\begin{aligned}&\quad \le \sum _{j_1,\dots , j_k=t!+1}^{|\Sigma _{t,t}|}\prod _{l=1}^k \frac{1}{\left( E_{j_l} \big | E_{j_l} \right) } \prod _{r=1}^{k-1} |\left. \left( {E_{j_r}}\right. \right| R(K)\left. \left| {E_{j_{r+1}}}\right. \right) | \cdot \Big \Vert \left. \left| {E_{j_1}}\right. \right) \!\! \left. \left( { E_{j_k}}\right. \right| \Big \Vert _{\diamond }. \end{aligned}$$

We now bound each of the factors in each term above. First, we compute the squared norm of $\left. \left| {E_j}\right. \right)$,

41 $$\begin{aligned} \left( E_j \big | E_j \right) = \sum _{r,l=1}^j A_{r,j} A_{l,j} \left( Q_{T_r} \big | Q_{T_l} \right) ^n = A_{j,j}^2 + \sum _{k,l< j} A_{r,j} A_{l,j} \left( Q_{T_k} \big | Q_{T_l} \right) ^n. \end{aligned}$$

Using Eqs. (32) and (33), we thus bound

42 $$\begin{aligned} \left( E_j \big | E_j \right)&\le \left( 1 + 2^{t^2+7t-n}\right) ^2 + (j^2-1) 4^{2t^2+10t-n} \nonumber \\&\le \left( 1 + 2^{t^2+7t-n}\right) ^2 + |\Sigma _{t,t}|^2 4^{2t^2+10t-n}\nonumber \\&\le 1 + 2^{31t^2 - 2n }, \end{aligned}$$

and in the same way

43 $$\begin{aligned} \left( E_j \big | E_j \right) \ge 1 - 2^{31t^2 - 2n }. \end{aligned}$$

Now we use that $n \ge 16t^2$. Letting $x:=2^{31t^2 - 2n }\in [0,\frac{1}{2}]$, the inequalities $1/(1-x)\le 1+2x$ and $1-2x\le 1/(1+x)$ hold. This leads to

44 $$\begin{aligned} \frac{1}{\left( E_j \big | E_j \right) }= 1+a_j\qquad \text {with}\qquad |a_j|\le 2^{32t^2-2n}. \end{aligned}$$

We now focus on the second factor,

45 $$\begin{aligned} |\left. \left( {E_i}\right. \right| R(K)\left. \left| {E_j}\right. \right) |\le \sum _{r=1}^i\sum _{l=1}^j |A_{r,i}A_{l,j}|\cdot \left| \left. \left( {Q_{T_r}^{\otimes n}}\right. \right| R(K)\left. \left| {Q_{T_l}^{\otimes n}}\right. \right) \right| . \end{aligned}$$

If for $\left. \left( {Q_{T_{r}}}\right. \right| R(K)\left. \left| {Q_{T_{l}}}\right. \right)$ one of the stochastic Lagrangian sub-spaces does not correspond to a permutation, Lemma 2 introduces a factor of $\eta _{K,t}$. If both correspond to a permutation, we redefine the factors in a way that leads to simpler expressions in the calculations used below. Namely, in this case we redefine $A_{r,i}$ and $A_{l,j}$ by multiplying it with 2. This is compensated by introducing a factor of $\frac{1}{4}$ and letting

46 $$\begin{aligned} {\bar{\eta }}_{K,t}:=\max \left\{ \frac{1}{4},\eta _{K,t}\right\} . \end{aligned}$$

We can do this as *i* and *j* do not correspond to permutations and hence $A_{r,j}$ and $A_{lj}$ are exponentially suppressed, which remains true after rescaling by 2. In this case, moreover, $r<t!+1\le i$ and $l< t!+1\le j$, so the factor $|A_{r,i}A_{l,j}|$ will be exponentially suppressed according to (32) and so this redefinition will not affect the asymptotic scaling in *n*.

We provide two bounds for $|\left. \left( {E_i}\right. \right| R(K)\left. \left| {E_j}\right. \right) |$ that will be used later on. We will use repeatedly that the diamond norm is multiplicative under the tensor product of superoperators [58, Thm. 3.49]. First, using (31), (33) and (28), we obtain

47 $$\begin{aligned}&|\left. \left( {E_i}\right. \right| R(K)\left. \left| {E_j}\right. \right) |\le \sum _{r=1}^i\sum _{l=1}^j |A_{r,i}A_{l,j}|\cdot \left| \left. \left( {Q_{T_r}^{\otimes n}}\right. \right| R(K)\left. \left| {Q_{T_l}^{\otimes n}}\right. \right) \right| \end{aligned}$$

48 $$\begin{aligned}&\quad \le {\bar{\eta }}_{K,t}(1+2^{8t^2-n}) \sum _{r=1}^i\sum _{l=1}^j 2^{24t^3-n|\dim N_r-\dim N_i|-n|\dim N_l-\dim N_j|-(n-1)|\dim N_l-\dim N_r|} \end{aligned}$$

49 $$\begin{aligned}&\quad \le {\bar{\eta }}_{K,t}(1+2^{8t^2-n})|\Sigma _{t,t}|^{2} 2^{25t^3-n|\dim N_j-\dim N_i|} \end{aligned}$$

50 $$\begin{aligned}&\quad \le {\bar{\eta }}_{K,t} (1+2^{8t^2-n}) 2^{31t^3-n|\dim N_j-\dim N_i|} , \end{aligned}$$

where we have used $2^{|\dim N_l-\dim N_r|}\le 2^t$ $\le 2^{t^3}$, and the fact that for the rescaled $A_{r,i}$, the inequality (31) implies

$$\begin{aligned} A_{r,i}\le 2^{11t^3 - |\dim N_r-\dim N_j|+1}\le 2^{12t^3 - |\dim N_r-\dim N_j|} \end{aligned}$$

for all *r*, *i*. Moreover, we have used the triangle inequality,

51 $$\begin{aligned}&|\dim N_r-\dim N_i|+|-\dim N_l+\dim N_j|+|\dim N_l-\dim N_r|\nonumber \\&\quad \ge |\dim N_r-\dim N_i-\dim N_l+\dim N_j+\dim N_l-\dim N_r|\nonumber \\&\quad = |\dim N_j - \dim N_i|, \end{aligned}$$

in the inequality (49).

The second bound follows from Eqs. (32) and (33), and we consider two cases. If $i\ne j$, then

52 $$\begin{aligned} |\left. \left( {E_i}\right. \right| R(K)\left. \left| { E_j}\right. \right) |&\le \sum _{r=1}^i\sum _{l=1}^j |A_{r,i}A_{l,j}|\cdot |\left. \left( {Q_{T_r}^{\otimes n}}\right. \right| R(K)\left. \left| {Q_{T_l}^{\otimes n}}\right. \right) |\nonumber \\&\le {\bar{\eta }}_{K,t}(1+2^{8t^2-n})|\Sigma _{t,t}|^2 2^{19t^2-n}\nonumber \\&\le {\bar{\eta }}_{K,t}(1+2^{8t^2-n})2^{25t^2-n}. \end{aligned}$$

Otherwise,

53 $$\begin{aligned} |\left. \left( {E_i}\right. \right| R(K)\left. \left| { E_i}\right. \right) |\le&\sum _{r=1}^i\sum _{l=1}^i |A_{r,i}A_{l,i}|\cdot |\left. \left( {Q_{T_r}^{\otimes n}}\right. \right| R(K)\left. \left| {Q_{T_l}^{\otimes n}}\right. \right) | \end{aligned}$$

54 $$\begin{aligned} \le&{\bar{\eta }}_{K,t}\left( |A_{i,i}|^2+(i^2-1) 2^{12t^2-n} \right) \end{aligned}$$

55 $$\begin{aligned} \le&{\bar{\eta }}_{K,t}\left( (1+2^{8t^2-n})^2+(1+2^{8t^2-n})2^{16t^2-n} \right) \end{aligned}$$

56 $$\begin{aligned} \le&{\bar{\eta }}_{K,t}(1+2^{16t^2-n})^3. \end{aligned}$$

In inequality (54), we have bounded the term $r=l=i$ using (33), and each of the other terms using (32). Moreover, in the inequalities (55) and (56) we use that $i\le |\Sigma _{t,t}|$, and

$$\begin{aligned} 1+2^{8t^2-n}\le (1+2^{8t^2-n})^2\le (1+2^{16t^2-n})^2. \end{aligned}$$

Lastly, we obtain from (31) and (27)

57 $$\begin{aligned} \Vert \left. \left| {E_i}\right. \right) \!\! \left. \left( {E_j}\right. \right| \Vert _{\diamond }&\le \sum _{r=1}^i\sum _{l=1}^j|A_{r,i}A_{l,j}|\cdot \left\| \left. \left| {Q_{T_r}^{\otimes n}}\right. \right) \!\! \left. \left( { Q_{T_l}^{\otimes n}}\right. \right| \right\| _{\diamond } \end{aligned}$$

58 $$\begin{aligned}&\le |\Sigma _{t,t}|^2 2^{24t^3-n|\dim N_r-\dim N_i|-n|\dim N_l-\dim N_j|+n(\dim N_l-\dim N_r)} \end{aligned}$$

59 $$\begin{aligned}&\le 2^{30t^3+n(\dim N_j-\dim N_i)}. \end{aligned}$$

We now start piecing these expressions together to bound (40). Eqs. (59) and (44) give

60 $$\begin{aligned}{} & {} \Vert [P_{{{\,\textrm{Cl}\,}}}\textrm{R}(K)]^k-P_{\textrm{H}}\Vert _{\diamond }\nonumber \\{} & {} \quad \le \left( 1+2^{32t^2-2n}\right) ^k\sum _{j_1,\dots , j_k=t!+1}^{|\Sigma _{t,t}|}2^{30t^3+n(\dim N_{j_k}-\dim N_{j_1})}\prod _{r=1}^{k-1}|\left. \left( { E_{j_r}}\right. \right| R(K)\left. \left| {E_{j_{r+1}}}\right. \right) |. \nonumber \\ \end{aligned}$$

To bound (60), we will bunch together the contribution of all terms whose sequence $\{j_1, \dots , j_k\}$ contains *l* changes. Moreover, we will treat differently the cases $l\le \lfloor t/2\rfloor$ and $l>\lfloor t/2\rfloor$. In the former case, we use (50) to get

61 $$\begin{aligned} \prod _{r=1}^{k-1}|\left. \left( {E_{j_r}}\right. \right| R(K)\left. \left| {E_{j_{r+1}}}\right. \right) | \le {\bar{\eta }}_{K,t}^{k-1}(1+2^{16t^2-n})^{3(k-1)}2^{ l 31t^3-n|\dim N_{j_k}-\dim N_{j_1}|}. \end{aligned}$$

In this case, the factor of $2^{n(\dim N_{j_k}-\dim N_{j_1})}$ coming from (59) is cancelled by the last factor of $2^{-n|\dim N_{j_k}-\dim N_{j_1}|}$.

In the latter case, we turn to (52) instead to obtain

$$\begin{aligned} \prod _{r=1}^{k-1}|\left. \left( {E_{j_r}}\right. \right| R(K)\left. \left| {E_{j_{r+1}}}\right. \right) | \le {\bar{\eta }}_{K,t}^{k-1}(1+2^{16t^2-n})^{3(k-1)} 2^{l 25 t^2 - ln}. \end{aligned}$$

Here, the exponential factor coming from (59) is cancelled by $2^{-ln}$ since $\dim N_{j_k}-\dim N_{j_1} \le \lfloor t/2\rfloor$. Counting the instances of sequences with *l* changes, we may put these considerations together to bound

$$\begin{aligned} \Vert [P_{{{\,\textrm{Cl}\,}}}\textrm{R}(K)]^k-P_{\textrm{H}}\Vert _{\diamond } \le&\left( 1+2^{32t^2-2n}\right) ^k\left( 1+2^{16t^2-n}\right) ^{3(k-1)}\\&\quad {\bar{\eta }}_{K,t}^{k-1} \Bigg [\sum _{l=0}^{\lfloor \frac{t}{2}\rfloor } {k\atopwithdelims ()l}|\Sigma _{t,t}|^{l+1}2^{l 31t^3}\\&+\sum _{l=\lfloor \frac{t}{2}\rfloor +1}^{k}{k\atopwithdelims ()l}|\Sigma _{t,t}|^{l+1}2^{(l-\lfloor \frac{t}{2}\rfloor )(25t^2-n)}2^{\lfloor \frac{t}{2}\rfloor 25t^2}\Bigg ]\\ \le&\left( 1+2^{32t^2-2n}\right) ^{4k}{\bar{\eta }}_{K,t}^{k-1} \Bigg [\frac{t}{2}{k\atopwithdelims ()\lfloor \frac{t}{2}\rfloor }|\Sigma _{t,t}|^{\lfloor \frac{t}{2}\rfloor +1}2^{\lfloor \frac{t}{2}\rfloor 31t^3} \\&+\sum _{l=1}^{k-\lfloor \frac{t}{2}\rfloor }{k\atopwithdelims ()l+\lfloor \frac{t}{2}\rfloor }|\Sigma _{t,t}|^{l+1+\lfloor \frac{t}{2}\rfloor }2^{l(25t^2-n)}2^{13t^3}\Bigg ]\\ {\mathop {\le }\limits ^{\ddagger }}&\left( 1+2^{32t^2-2n}\right) ^{4k}{\bar{\eta }}_{K,t}^{k-1}\Bigg [ 2^{32t^4+t\log (k)}\\&+k^{\lfloor \frac{t}{2}\rfloor }|\Sigma _{t,t}|^{1+\lfloor \frac{t}{2}\rfloor }2^{13t^3}\sum _{l=0}^{k}{k\atopwithdelims ()l}|\Sigma _{t,t}|^{l}2^{l(25t^2-n)}\Bigg ]\\ \le&\left( 1+2^{32t^2-2n}\right) ^{4k}{\bar{\eta }}_{K,t}^{k-1}\Bigg [ 2^{32t^4+t\log (k)}\\&\quad +2^{18t^3+\log (k)t}\left( 1+2^{28t^2-n}\right) ^{k}\bigg ]\\ { \le }&\left( 1+2^{32t^2-2n}\right) ^{4k}\left( 1+2^{28t^2-n}\right) ^{k}\\&\quad 2^{t\log (k)}{\bar{\eta }}_{K,t}^{k-1}\Bigg [ 2^{32t^4}+2^{18t^3}\bigg ], \end{aligned}$$

where we have used in $\ddagger$ that

$$\begin{aligned} {k\atopwithdelims ()l+\lfloor \frac{t}{2}\rfloor }&=\frac{(k)!}{(k-l-\lfloor \frac{t}{2}\rfloor )!(l+\lfloor \frac{t}{2}\rfloor )!}\\&\le (k-l-\big \lfloor \frac{t}{2}\big \rfloor +1)\dots (k-l)\frac{k!}{(k-l)!l!}\\&\le k^{\lfloor \frac{t}{2}\rfloor }{k\atopwithdelims ()l}. \end{aligned}$$

Finally, noting that $2^{32t^4}+2^{18t^3}\le 2^{33t^4}$ for all positive integers *t*, we obtain the bound

62 $$\begin{aligned} \Vert \textrm{M}_t(\sigma _{k}) - P_{\textrm{H}} \Vert _{\diamond } \le 2^{33t^4+t\log (k)}\left( 1+2^{32t^2-n}\right) ^{5k}{\bar{\eta }}_{K,t}^{k-1}, \end{aligned}$$

where ${\bar{\eta }}_{K,t}$ is bounded by Lemma 2. Taking the logarithm and using the inequality $\log (1+x)\le x$ repeatedly, this implies Theorem 1. $\square$

With the above bound, we can also prove Corollary 1.

### Proof of Corollary 1

Consider the self-adjoint superoperator $A:=P_{{{\,\textrm{Cl}\,}}}R(K)P_{{{\,\textrm{Cl}\,}}}$. As $P_{{{\,\textrm{Cl}\,}}}$ is a projector, we have with Eq. (24)

63 $$\begin{aligned} (A-P_\textrm{H})^k = A^k - P_\textrm{H}= \left[ P_{{{\,\textrm{Cl}\,}}} R(K)\right] ^k - P_\textrm{H}= \textrm{M}_t(\sigma _{k}) - P_{\textrm{H}}. \end{aligned}$$

Using norm inequality between operator and diamond norm Eq. (12) and the previous result Eq. (62), we find

64 $$\begin{aligned} ||A-P_H||^{k}_{\infty }=||(A-P_H)^k||_{\infty }\le & {} 2^{nt/2}\Vert \textrm{M}_t(\sigma _{k}) - P_{\textrm{H}} \Vert _{\diamond }\nonumber \\\le & {} 2^{33t^4+t\log (k)+nt/2}\left( 1+2^{32t^2-n}\right) ^{5k}{\bar{\eta }}_{K,t}^{k-1}. \end{aligned}$$

Taking the *k*-th square root of the expresion above, we obtain a sequence of infinitely many bounds for $||A-P_H||_{\infty }$ which converges as $k\rightarrow \infty$. That limit gives

65 $$\begin{aligned} ||A-P_H||_{\infty }\le \left( 1+2^{32t^2-n}\right) ^{5}{\bar{\eta }}_{K,t}. \end{aligned}$$

Combined with Ref. [24, Lem. 4], Eq. (65) implies the result. $\square$

The bound in Eq. (62) also suffices to prove Proposition 1:

### Proof of Proposition 1

The proof follows exactly as the proof of Theorem 1, but with the factor 7/8 instead of ${\bar{\eta }}_{K,t}$ (compare Lemma 13). Using $\log _2(7/8)\le -0.19$ the result can be checked. $\square$

## Convergence to Higher Moments of the Clifford Group

In this section, we aim to prove:

### Theorem 2

(Local random Clifford designs). Let $n\ge 12t$, then a local random Clifford circuit of depth $O(n\log ^{-2}(t)t^{8}(2nt+\log (1/\varepsilon )))$ constitutes a relative $\varepsilon$-approximate Clifford *t*-design.

The proof of Theorem 2 follows a well-established strategy [24, 59] in a sequence of lemmas. For the sake of readibility, the proofs of these lemmas have been moved to Sect. 6.4. Given a measure $\nu$ on the Clifford group ${{\,\textrm{Cl}\,}}(n)$, recall that its *t*-th moment operator was defined as

$$\begin{aligned} \textrm{M}_t(\nu ) := \int _{\mathrm{Cl(2^n)}} {{\,\textrm{Ad}\,}}_U^{\otimes t}\textrm{d}\nu (U). \end{aligned}$$

The idea of the proof is that if $\textrm{M}_t(\nu )$ is close to the moment operator $\textrm{M}_t(\mu _{{{\,\textrm{Cl}\,}}})\equiv P_{{{\,\textrm{Cl}\,}}}$ of the uniform (Haar) measure $\mu _{\textrm{Cl}}$ on the Clifford group, $\nu$ is an approximate Clifford design. However, we have seen that there are different notions of closeness. We define its deviation in (superoperator) *spectral norm* as

$$\begin{aligned} g_{\textrm{Cl}}(\nu ,t) := \left\| \textrm{M}_t(\nu ) - \textrm{M}_t(\mu _{{{\,\textrm{Cl}\,}}}) \right\| _{\infty }. \end{aligned}$$

Then, we prove the following lemma in Sect. 6.4.

### Lemma 5

(Relative $\varepsilon 2^{2tn}$-approximate Clifford *t*-designs). Suppose that $0 \le \varepsilon < 1$ is such that $g_{\textrm{Cl}}(\nu ,t)\le \varepsilon$. Then, $\nu$ is a relative $\varepsilon 2^{2tn}$-approximate Clifford *t*-design.

Recall that we have defined the measure $\sigma _G$ on the Clifford group ${{\,\textrm{Cl}\,}}(n)$ in Def. 4 by randomly drawing from a 2-local Clifford gate set *G* and applying it to a random qubit *i*, or to a pair of adjacent qubits $(i,i+1)$, respectively. For this measure, we show that it fulfills the assumptions of Lemma 5:

### Proposition 2

(Clifford expander bound). Let $\sigma _G$ be as in Def. 4 and $n \ge 12t$. Then, $g_\textrm{Cl}(\sigma _G,t) \le 1 - c(G) n^{-1} \log ^2(t) t^{-8}$ for some constant $c(G)>0$.

We will prove Proposition 2 in the end of this section. From this, Theorem 2 follows as a direct consequence:

### Proof of Theorem 2

First, note that $g_{{{\,\textrm{Cl}\,}}}(\nu ^{*k},t)=g_{{{\,\textrm{Cl}\,}}}(\nu ,t)^k$ for all probability measures $\nu$ on the Clifford group. This can be easily verified using the observation

66 $$\begin{aligned} \textrm{M}_t(\mu _{{{\,\textrm{Cl}\,}}}) \textrm{M}_t(\nu ) = \textrm{M}_t(\nu ) \textrm{M}_t(\mu _{{{\,\textrm{Cl}\,}}}) = \textrm{M}_t(\mu _{{{\,\textrm{Cl}\,}}}). \end{aligned}$$

Hence, combining the bound given by Proposition 2 and Lemma 5, we find that the *k*-step random walk $\sigma _G^{*k}$ is a $\varepsilon$-approximate Clifford *t*-design, if we choose $k = O\left( n \log ^{-2}(t) t^8\left( 2nt + \log (1/\varepsilon ) \right) \right)$. $\square$

For the sake of readibility, let us from now on drop the dependence on *G* and write $\sigma \equiv \sigma _G$. In order to prove Proposition 2, we use a reformulation of $g(\sigma ,t)$ based on the following observation. Since *G* is closed under taking inverses, the moment operator $\textrm{M}_t(\sigma )$ is self-adjoint with respect to the Hilbert-Schmidt inner product. Due to $\sigma$ being a probability measure, its largest eigenvalue is 1 with eigenspace corresponding to the operator subspace which is fixed by the adjoint action ${{\,\textrm{Ad}\,}}(g^{\otimes t})$ of all generators [59]. Equivalently, this is the subspace of operators which commute with any generator $g^{\otimes t}$. However, any operator commuting with all generators also commutes with every element in the Clifford group ${{\,\textrm{Cl}\,}}(n)$ and vice versa. Hence, this subspace is nothing but the Clifford commutant ${{\,\textrm{Cl}\,}}(n)'$ with projector $P_{{{\,\textrm{Cl}\,}}} {:=} \textrm{M}_t(\mu _{{{\,\textrm{Cl}\,}}})$. Thus, the spectral decomposition is

67 $$\begin{aligned} \textrm{M}_t(\sigma ) = P_{{{\,\textrm{Cl}\,}}} + \sum _{r \ge 2} \lambda _r(\textrm{M}_t(\sigma )) \Pi _r, \end{aligned}$$

where $\lambda _r(X)$ denotes the *r*-th largest eigenvalue of a normal operator *X*. Hence, we find

68 $$\begin{aligned} g(\sigma ,t)= \left\| \textrm{M}_t(\sigma ) - P_{{{\,\textrm{Cl}\,}}} \right\| _{\infty } = \lambda _*\left( \textrm{M}_t(\sigma )\right) := \max \left\{ \lambda _2\left( \textrm{M}_t(\sigma )\right) , |\lambda _\textrm{min}\left( \textrm{M}_t(\sigma )\right) | \right\} ,\nonumber \\ \end{aligned}$$

where $\lambda _\textrm{min}\left( \textrm{M}_t(\sigma )\right)$ is the smallest eigenvalues of $\textrm{M}_t(\sigma )$. We continue by arguing that it sufficient to consider the case when $\lambda _*\left( \textrm{M}_t(\sigma )\right) = \lambda _2\left( \textrm{M}_t(\sigma )\right) > 0$.

To this end, consider the linear operator $T_{\sigma }:L^2({{\,\textrm{Cl}\,}}(n))\rightarrow L^2({{\,\textrm{Cl}\,}}(n))$ given as

69 $$\begin{aligned} T_{\sigma }f(g):=\int f(h^{-1}g)\textrm{d}\sigma (h). \end{aligned}$$

This is the (Hermitian) averaging operator with respect to $\sigma$ on the group algebra $L^2({{\,\textrm{Cl}\,}}(n))$. The largest eigenvalue of $T_\sigma$ is $\lambda _1(T_\sigma )=1$ and its eigenspace corresponds to the trivial representation. By Ref. [60, Lem. 1], its smallest eigenvalue is lower bounded by

70 $$\begin{aligned} \lambda _\textrm{min}(T_\sigma ) \ge -1 + 2 \sigma (\mathbbm {1}) = -1 + \frac{2}{|G|}, \end{aligned}$$

where $\sigma (\mathbbm {1}) \equiv \sigma (\{\mathbbm {1}\})=1/|G|$ is the probability of drawing the identity. According to the Peter-Weyl theorem, the spectrum of $\textrm{M}_t(\sigma )$ is exactly the spectrum of the restriction of $T_\sigma$ to the irreducible representations that appear in the representation $U\mapsto {{\,\textrm{Ad}\,}}_U^{\otimes t}$. In particular, we find $\lambda _\textrm{min}(\textrm{M}_t(\sigma )) \ge -1 + \frac{2}{|G|}$. Let us assume that $\lambda _*\left( \textrm{M}_t(\sigma )\right) = |\lambda _\textrm{min}\left( \textrm{M}_t(\sigma )\right) |$. Then, $g(\sigma ,t) \le 1 - 2/|G| < 1$ and hence we can argue as in the proof of Thm. 2 to show that local random Clifford circuits form relative $\varepsilon$-approximate Clifford *t*-designs in depth $O(2nt + \log (1/\varepsilon ))$.

Therefore, we consider the more relevant case when $\lambda _*\left( \textrm{M}_t(\sigma )\right) = \lambda _2\left( \textrm{M}_t(\sigma )\right) > 0$ in the following, this is

71 $$\begin{aligned} g(\sigma ,t)= \left\| \textrm{M}_t(\sigma ) - P_{{{\,\textrm{Cl}\,}}} \right\| _{\infty } = \lambda _2\left( \textrm{M}_t(\sigma )\right) . \end{aligned}$$

Since $\textrm{M}_t(\sigma )$ is self-adjoint, we can interpret it as an Hamiltonian on the Hilbert space $L( ({\mathbb {C}}^2)^{\otimes nt})$. In this light, it will turn out to be useful to recast Eq. (71) as the spectral gap of a suitable family of *local Hamiltonians* with vanishing ground state energy:

72 $$\begin{aligned} H_{n,t}:=n \left( \textrm{id}- \textrm{M}_t(\sigma ) \right) = \sum _{i=1}^n h_{i,i+1}, \quad \text {with}\quad h_{i,i+1}:=\frac{1}{|G|}\sum _{g\in G}\left( \textrm{id}- {{\,\textrm{Ad}\,}}(g_{i,i+1}^{\otimes t})\right) .\nonumber \\ \end{aligned}$$

Let us summarize these findings in the following lemmas.

### Lemma 6

(Spectral gap). Let $\sigma$ be as in Def. 4 and $H_{n,t}$ the Hamiltonian from Eq. (72). It holds that

73 $$\begin{aligned} g(\sigma ,t)=1-\frac{\Delta (H_{n,t})}{n}. \end{aligned}$$

### Lemma 7

(Ground spaces). The Hamiltonians $H_{n,t}$ are positive operators with ground state energy 0. The ground space is given by the Clifford commutant

74 $$\begin{aligned} {{\,\textrm{Cl}\,}}(n)'={{\,\textrm{span}\,}}\left\{ r(T)^{\otimes n} \; \big | \; T \in \Sigma _{t,t} \right\} , \end{aligned}$$

where $\Sigma _{t,t}$ is the set of stochastic Lagrangian sub-spaces of ${\mathbb {Z}}_2^t\oplus {\mathbb {Z}}_2^t$.

In the remainder of this section, we will prove the existence of a uniform lower bound on the spectral gap of $H_{n,t}$. In combination with Lemma 6 and Lemma 5 this will imply Theorem 2. While it is highly non-trivial to show spectral gaps in the thermodynamic limits, we can use the fact that $H_{n,t}$ is *frustration-free* (compare Lemma 7). This allows us to apply the powerful *martingale method* pioneered by Nachtergaele [61].

### Lemma 8

(Lower bound to spectral gap) Let the Hamiltonian $H_{n,t}$ be as in Eq. (72) and assume that $n\ge 12t$. Then, $H_{n,t}$ has a spectral gap satisfying

75 $$\begin{aligned} \Delta (H_{n,t})\ge \frac{\Delta (H_{12t,t})}{48t}. \end{aligned}$$

### Proof of Proposition 2

We can now combine the bound in (75) with any lower bound on the spectral gap independent of *t*. To this end, we make again use of the averaging operator $T_{\sigma }:L^2({{\,\textrm{Cl}\,}}(n))\rightarrow L^2({{\,\textrm{Cl}\,}}(n))$ introduced in Eq. (69) before. By Ref. [60, Cor. 1] we have that

76 $$\begin{aligned} \lambda _2(T_\sigma )\le 1-\frac{\eta }{d^2}, \end{aligned}$$

where $\eta$ is the probability of the least probable generator (here 1/|*G*|*n*) and *d* is the diameter of the associated Cayley graph (given in Ref. [62] as $d=O(n^3/\log (n))$.

Since the representation $U\mapsto {{\,\textrm{Ad}\,}}_U^{\otimes t}$ contains a trivial component, the second largest eigenvalue of $\textrm{M}_t(\sigma )$ can be at most $\lambda _2(T_\sigma )$. Thus, $H_{n,t}$ has a gap of at least $\eta /d^2$. Finally, by Lemma 8 it follows that

77 $$\begin{aligned} \Delta (H_{n,t})\ge \frac{\Delta (H_{12t,t})}{48t} \ge c(G) t^{-8}\log (t)^2, \end{aligned}$$

for a constant *c*(*G*). We note that the applicability of Ref. [60, Cor. 1] to random walks on the Clifford group has also been observed in Ref. [9]. $\square$

We can combine Theorem 2 and Theorem 1 to obtain the following corollary:

### Corollary 2

(Local random unitary design). Let $K \in U(2)$ be a non-Clifford gate and let $G \subset {{\,\textrm{Cl}\,}}(4)$ be a closed, generating set. There are constants $C''_1(K,G), C''_2(K), C''_3(K)$ such that whenever

$$\begin{aligned} m \ge C''_1(K,G)n \log ^{-2}(t) t^8\left( 2nt + \log (1/\varepsilon ) \right) \ \text { and }\ k\ge C''_2(K)\log ^2(t)(t^{4}+t\log (1/\varepsilon )), \end{aligned}$$

the local random circuit $\sigma _{k,m}$, defined in (6), is an $\varepsilon$-approximate unitary *t*-design for all $n\ge C''_3(K)t^2$.

### Proof

Consider the superoperator

78 $$\begin{aligned} \textrm{M}_t(\sigma _{k,m}) = \int _{U(2^n)} {{\,\textrm{Ad}\,}}(U^{\otimes t})\,\textrm{d}\sigma _{k,m}(U)=\underbrace{ \textrm{M}_t(\sigma ^{*m})\textrm{R}(K)\dots \textrm{M}_t(\sigma ^{*m})\textrm{R}(K)}_{k \text { times}}, \end{aligned}$$

where $\sigma ^{*m}$ denotes the probability measure of a depth *m* local random walk on the Clifford group (cp. Def. 4). We would like to bound the difference between the Haar random *t*-th moment operator $\textrm{M}_t(\mu _\textrm{H})=:P_\textrm{H}$ and $\textrm{M}_t(\sigma _{k,m})$. Notice the following standard properties of $P_\textrm{H}$:

79 $$\begin{aligned} P_{\textrm{H}}\textrm{M}_t(\nu )=\textrm{M}_t(\nu )P_{\textrm{H}}=P_{\textrm{H}},\qquad \text {and} \qquad P_{\textrm{H}}^{\dagger }=P_{\textrm{H}}, \end{aligned}$$

for any probability measure $\nu$ on $U(2^n)$. In particular, we have that $P_{\textrm{H}}$ is an orthogonal projector. As in the last section, we make use of the spectral decomposition in Eq. (67) to decompose $\textrm{M}_t(\sigma ^{*k})$ as follows:

80 $$\begin{aligned} \textrm{M}_t(\sigma _{k,m}) - P_{\textrm{H}}&= \left[ \textrm{M}_t(\sigma ^{*m})\textrm{R}(K) \right] ^k - P_{\textrm{H}}\nonumber \\&= \left[ \bigg (P_{{{\,\textrm{Cl}\,}}} + \sum _{i\ge 2}\lambda ^m_i \Pi _i \bigg )\textrm{R}(K) \right] ^k - P_{\textrm{H}}. \end{aligned}$$

Recall the shorthand notation $P_{{{\,\textrm{Cl}\,}}}:= \textrm{M}_t(\mu _{{{\,\textrm{Cl}\,}}})$. Using the triangle inequality and the inequality (12), this implies

81 $$\begin{aligned} \left\| \textrm{M}_t(\sigma _{k,m})-P_{\textrm{H}} \right\| _\diamond&\le \bigl \Vert [P_{{{\,\textrm{Cl}\,}}}\textrm{R}(K)]^k -P_{\textrm{H}} \bigr \Vert _\diamond + 2^{2tn}\sum _{l=1}^{k}{k\atopwithdelims ()l}\lambda _{2}^{lm}\nonumber \\&\le \bigl \Vert [P_{{{\,\textrm{Cl}\,}}}\textrm{R}(K)]^k -P_{\textrm{H}} \bigr \Vert _\diamond + k2^{2tn+1}\lambda _2^m. \end{aligned}$$

Note that we bounded the second largest eigenvalue $\lambda _2$ of $\textrm{M}_t(\sigma )$ in Proposition 2. We can now combine Proposition 2 with (62) to obtain:

82 $$\begin{aligned} \Vert \textrm{M}_t(\sigma _{k,m}) - P_{\textrm{H}} \Vert _{\diamond } \le k2^{2tn+1}\lambda _2^m+ 2^{33t^4+t\log (k)}\left( 1+2^{32t^2-n}\right) ^{5k}{\bar{\eta }}_{K,t}^k. \end{aligned}$$

$$\square $$

## Singling out the Clifford Group

There are a number of ways to motivate the construction of approximate unitary *t*-designs from random Clifford circuits. From a practical point of view, Clifford gates are often comparatively easy to implement, in particular in fault-tolerant architectures. In this section, we point out that Refs. [38, 39] together imply that the Clifford groups are also mathematically distinguished. We formulate this observation as Proposition 3: The finite case follows from the recently obtained classification of finite unitary subgroups forming *t*-designs, so-called *unitary* *t*-*groups*, by [38] building on earlier results by [55]. The infinite case is a corollary of a theorem about universality of finitely generated subgroups by [39].

This section is independent from the rest of the paper and has the sole purpose of highlighting the results in Refs. [38, 39, 55] and explicitly formulate their combined implications for the generation of unitary *t*-designs. Moreover, it might serve as an intuitive justification for the usefulness and omnipresence of Clifford unitaries in random circuit constructions.

For any subgroup $G\subseteq \textrm{U}(d)$, we let

$$\begin{aligned} {\overline{G}}:=\{\det (U^\dagger )U\,|\, U\in G\}\subseteq {{\,\textrm{SU}\,}}(d). \end{aligned}$$

Notice that ${\overline{G}}$ is a unitary *t*-design if and only if *G* is.

Proposition 3 refers to *t*-designs generated by *finite gate sets*, which we define now. The starting point is a Hilbert space $({\mathbb {C}}^q)^{\otimes r}$ for some *r*. A finite gate set is a finite subset

$$\begin{aligned} {\mathcal {G}}\subset {{\,\textrm{SU}\,}}\big (({\mathbb {C}}^q)^{\otimes r}\big ). \end{aligned}$$

We will denote by ${\mathcal {G}}_n$ the subgroup of ${{\,\textrm{SU}\,}}\big (({\mathbb {C}}^q)^{\otimes n}\big )$ generated by elements of ${\mathcal {G}}$ acting on any *r* tensor factors (here $r\le n$). The number *q* is called the *local dimension* of ${\mathcal {G}}$.

### Proposition 3

(Singling out the Clifford group [38, 39, 55]). Let $t\ge 2$, and let ${\mathcal {G}}$ be a finite gate set with local dimension $q\ge 2$. Assume that (1) either all ${\mathcal {G}}_n$ are finite or they are all infinite, and (2) there is an $n_0$ such that for all $n\ge n_0$, ${\mathcal {G}}_n$ is a unitary *t*-design.

Then, one of the following cases apply:

(i) If $t=2$, we have either *q* prime and ${\mathcal {G}}_n$ is isomorphic to a subgroup of the Clifford group ${\overline{{{\,\textrm{Cl}\,}}}}(q^n)$, *or* ${\mathcal {G}}_n$ is dense in $\textrm{SU}(q^n)$,
(ii) If $t=3$, we have *either* $q=2$ and ${\mathcal {G}}_n$ is isomorphic to the full Clifford group ${\overline{{{\,\textrm{Cl}\,}}}}(2^n)$ *or* ${\mathcal {G}}_n$ is dense in $\textrm{SU}(q^n)$,
(iii) If $t \ge 4$ then ${\mathcal {G}}_n$ is dense in $\textrm{SU}(q^n)$.

Note that a finitely generated infinite subgroup of ${{\,\textrm{SU}\,}}(d)$ is always dense in some compact Lie subgroup (cp. [39, Fact 2.6]). In particular, it inherits a Haar measure from this Lie subgroup which allows for a definition of unitary *t*-design.

*a. Finite case.* In the classification in Ref. [38], the non-existence of finite unitary *t*-groups was shown for $t \ge 4$ (and dimension $d>2$). Already the case $t=3$ is very restrictive, since the authors arrive at the following result:

### Lemma 9

(Ref. [38, Thm. 4]). Suppose $d\ge 5$ and consider a finite subgroup $H < {{\,\textrm{SU}\,}}(d)$ which is a unitary 3-design. Then, *H* is either one of finitely many exceptional cases or $d=2^n$ and *H* is isomorphic to the Clifford group ${\overline{{{\,\textrm{Cl}\,}}}}(2^n)$.

This establishes the finite version of (*ii*), the $t=3$ case.

The classification of unitary 2-designs is however more involved, it includes certain irreducible representations of finite unitary and symplectic groups (compare [38, Thm. 3 Lie-type case]), and a finite set of exceptions. The exceptions can be ruled out in the same way as above.

The former, the Lie-type cases, happen in dimensions $(3^n\pm 1)/2$ and $(2^n+(-1)^n)/3$. There is no *q* for which there exists an $n_0$ such that for all $n\ge n_0$ there exists an $m\in {\mathbb {N}}$ satisfying either

$$\begin{aligned} q^n = (3^m\pm 1)/2 \qquad \text {or}\qquad q^n = (2^m+(-1)^m)/3. \end{aligned}$$

Thus, the assumptions of Prop. 3 rule these out. This establishes the finite version of (*i*).

*b. Infinite case.* Define the commutant for a set $S\subset {{\,\textrm{SU}\,}}(d)$ of the adjoint action as

$$\begin{aligned} {{\,\textrm{Comm}\,}}(\textrm{Ad}_S) :=\left\{ L\in {{\,\textrm{End}\,}}\left( {\mathbb {C}}^{d\times d}\right) \,\big | \, [{{\,\textrm{Ad}\,}}_g,L]=0\;\;\forall g\in S\right\} . \end{aligned}$$

We show that the second case can be reduced to Cor. 3.5 from Ref. [39] applied to the simple Lie group ${{\,\textrm{SU}\,}}(d)$.

### Lemma 10

([39, Cor. 3.5]). Given a finite set $G\subset {{\,\textrm{SU}\,}}(d)$ such that ${\mathcal {G}} = \langle G \rangle$ is infinite. Then, the group ${\mathcal {G}}$ is dense in ${{\,\textrm{SU}\,}}(d)$ if and only if

83 $$\begin{aligned} {{\,\textrm{Comm}\,}}({{\,\textrm{Ad}\,}}_{{\mathcal {G}} })\cap {{\,\textrm{End}\,}}({{\mathfrak {s}}}{{\mathfrak {u}}}(d))=\{\lambda \, \textrm{id}_{{{\mathfrak {s}}}{{\mathfrak {u}}}(d)} \,|\,\lambda \in {\mathbb {R}}\}. \end{aligned}$$

Recall that a subgroup ${\mathcal {G}}\subseteq U(d)$ is a unitary 2-group if and only if ${{\,\textrm{Comm}\,}}(U\otimes U|U\in {\mathcal {G}})={{\,\textrm{Comm}\,}}(U\otimes U|U\in {{\,\textrm{U}\,}}(d))={{\,\textrm{span}\,}}(\mathbbm {1},{\mathbb {F}})$, where ${\mathbb {F}}$ denotes the flip of two tensor copies (see also App. A). Let us denote the partial transpose on the second system of a linear operator $A\in L({\mathbb {C}}^{d}\otimes {\mathbb {C}}^{d})$ by $A^{\Gamma }$. Then, one can easily verify that $\Gamma$ induces a vector space isomorphism between ${{\,\textrm{Comm}\,}}(U\otimes U|U\in {\mathcal {G}})$ and ${{\,\textrm{Comm}\,}}(U\otimes {{\overline{U}}}|U\in {\mathcal {G}})$. The image of the basis $\{\mathbbm {1},{\mathbb {F}}\}$ is readily computed as

84 $$\begin{aligned} \mathbbm {1}^{\Gamma } = \mathbbm {1}, \quad \quad \quad {\mathbb {F}}^{\Gamma } = d \left. \left| {\Omega }\right. \right\rangle \!\! \left. \left\langle {\Omega }\right. \right| , \end{aligned}$$

where $\left. \left| {\Omega }\right. \right\rangle = d^{-1/2}\sum _{i=1}^d \left. \left| {ii}\right. \right\rangle$ is the maximally entangled state vector. Next, we use that $U\otimes {\overline{U}}={{\,\textrm{mat}\,}}({{\,\textrm{Ad}\,}}_U)$ is the matrix representation of ${{\,\textrm{Ad}\,}}_U=U\cdot U^\dagger$ with respect to the basis $E_{i,j}=\left. \left| {i}\right. \right\rangle \!\! \left. \left\langle {j}\right. \right|$ of $L({\mathbb {C}}^d)$. Thus, we have ${{\,\textrm{Comm}\,}}({{\,\textrm{Ad}\,}}_{{\mathcal {G}}}) \simeq {{\,\textrm{Comm}\,}}(U\otimes {\overline{U}}|U\in {\mathcal {G}})$ as algebras. Pulling the above basis of ${{\,\textrm{Comm}\,}}(U\otimes {\overline{U}}|U\in {\mathcal {G}})$ back to ${{\,\textrm{Comm}\,}}({{\,\textrm{Ad}\,}}_{{\mathcal {G}}})$, we then find:

85 $$\begin{aligned} {{\,\textrm{mat}\,}}^{-1}(\mathbbm {1}) = \textrm{id}_{L({\mathbb {C}}^d)}, \quad {{\,\textrm{mat}\,}}^{-1}(\left. \left| {\Omega }\right. \right\rangle \!\! \left. \left\langle {\Omega }\right. \right| ) = {{\,\textrm{Tr}\,}}(\bullet ) \textrm{id}_{L({\mathbb {C}}^d)}. \end{aligned}$$

Hence, we have shown that any element in ${{\,\textrm{Comm}\,}}({{\,\textrm{Ad}\,}}_{{\mathcal {G}}})$ is a linear combination of these two maps. However, by restricting to ${{\mathfrak {s}}}{{\mathfrak {u}}}(d)$, the second map becomes identically zero, thus we have

86 $$\begin{aligned} {{\,\textrm{Comm}\,}}({{\,\textrm{Ad}\,}}_{{\mathcal {G}} })\cap {{\,\textrm{End}\,}}({{\mathfrak {s}}}{{\mathfrak {u}}}(d))=\{\lambda \, \textrm{id}_{{{\mathfrak {s}}}{{\mathfrak {u}}}(d)} \,|\,\lambda \in {\mathbb {R}}\}. \end{aligned}$$

By Lemma 10, this shows that any finitely generated infinite unitary 2-group ${\mathcal {G}}\le {{\,\textrm{SU}\,}}(d)$ is dense in ${{\,\textrm{SU}\,}}(d)$. Since any unitary *t*-group is in particular a 2-group, this is also true for any $t>2$.

## Proofs

### Proof of overlap lemmas

In this section, we prove three technical lemmas which are needed throughout this paper. These lemmas give bounds on the overlaps of the operators $Q_T^{\otimes n}$ and hence quantify how far this basis is from an orthonormal basis of the commutant of the Clifford tensor power representation, i.e., for $\textrm{range}\ P_{{{\,\textrm{Cl}\,}}}$.

#### Lemma 3

(Diamond norm bound). Consider $T_1,T_2\in \Sigma _{t,t}$ and denote with $N_1,N_2$ their respective defect spaces. Then, it holds that

27 $$\begin{aligned} \left\| \left. \left| {Q_{T_1}}\right. \right) \!\! \left. \left( {Q_{T_2}}\right. \right| \right\| _\diamond&\le 2^{\dim N_2-\dim N_1}, \end{aligned}$$

28 $$\begin{aligned} |\left( Q_{T_1} \big | Q_{T_2} \right) |&\le 2^{-|\dim N_1-\dim N_2|}. \end{aligned}$$

#### Proof

First, recall that $Q_T:= 2^{-t/2}r(T)$. Then, we make use of the following elementary bound on the diamond norm of rank one superoperator $\left. \left| {A}\right. \right) \!\! \left. \left( {B}\right. \right|$:

87 $$\begin{aligned} \left\| \left. \left| {A}\right. \right) \!\! \left. \left( {B}\right. \right| \right\| _\diamond&= \sup _{\left\| X \right\| _{1}=1} \left\| A\otimes {{\,\textrm{Tr}\,}}_1\left( B\otimes \mathbbm {1}X\right) \right\| _{1} \nonumber \\&{\mathop {\le }\limits ^{\dagger }} \left\| A \right\| _{1} \sup _{\left\| X \right\| _{1}=1} \left\| B\otimes \mathbbm {1}X \right\| _{1} \nonumber \\&{\mathop {=}\limits ^{\ddagger }} \left\| A \right\| _{1} \left\| B\otimes \mathbbm {1} \right\| _\infty \nonumber \\&= \left\| A \right\| _{1} \left\| B \right\| _\infty . \end{aligned}$$

Here, we have used in $\dagger$ that the partial trace is a contraction w.r.t. $\left\| \cdot \right\| _{1}$ and in $\ddagger$ a version of the duality between trace and spectral norm [63]. Given stochastic Lagrangians $T_1$ and $T_2$ with defect spaces $N_1$ and $N_2$, we thus find using Lem. 1:

88 $$\begin{aligned} \left\| \left. \left| {Q_{T_1}}\right. \right) \!\! \left. \left( {Q_{T_2}}\right. \right| \right\| _\diamond \le 2^{-t} \left\| r(T_1) \right\| _{1}\left\| r(T_2) \right\| _\infty = 2^{\dim N_2 - \dim N_1}. \end{aligned}$$

To prove 2., we use Ref. [42, Eq. (4.25)] and that the transpose does not change the dimension of the corresponding defect subspace. Moreover, we assume w.l.o.g. that $\dim N_2\ge \dim N_1$. We have

89 $$\begin{aligned} |\left( Q_{T_1} \big | Q_{T_2} \right) |= 2^{-t}|{{\,\textrm{Tr}\,}}[r(T_1)r(T_2)^T]|=2^{-t+\dim ( N_1\cap N_2)}|{{\,\textrm{Tr}\,}}[r(T)]| \end{aligned}$$

where *r*(*T*) is described by a stochastic orthogonal and a defect space $N^{\perp }_1\cap N_2+N_1$. Hence, we obtain (together with Hölder’s inequality):

90 $$\begin{aligned} |\left( Q_{T_1} \big | Q_{T_2} \right) |\le 2^{-t+\dim ( N_1\cap N_2)}2^{t-\dim (N_1^{\perp }\cap N_2+N_1)}. \end{aligned}$$

Using $N\subseteq N^{\perp }$ for all defect spaces and the general identity $\dim (V+W)=\dim V+\dim W-\dim (V\cap W)$, this yields

91 $$\begin{aligned} |\left( Q_{T_1} \big | Q_{T_2} \right) | \le 2^{\dim (N_1\cap N_2)-\dim N_1}\le 2^{\dim N_2-\dim N_1}. \end{aligned}$$

$$\square $$

Next, we define a *frame operator* associated to the basis $Q_T^{\otimes n}$. If the basis was orthogonal, this frame operator would simply be the projector $P_{{{\,\textrm{Cl}\,}}}$ onto the Clifford commutant.

#### Definition 9

(*Clifford frame operator*). We define the Clifford frame operator of the basis $Q_T^{\otimes n}$ as

92 $$\begin{aligned} S_{{{\,\textrm{Cl}\,}}} := \sum _{T\in \Sigma _{t,t}} \left. \left| {Q_T}\right. \right) \!\! \left. \left( {Q_T}\right. \right| ^{\otimes n}. \end{aligned}$$

Hence, a quantifier for the orthogonality of the $Q_T^{\otimes n}$ basis is the distance of $S_{{{\,\textrm{Cl}\,}}}$ to the projector $P_{{{\,\textrm{Cl}\,}}}$. As we prove in Lem. 12, we have $P_{{{\,\textrm{Cl}\,}}} \approx S_{{{\,\textrm{Cl}\,}}}$ in spectral norm and we will use this result later in the proof of Lem. 8. In order to show this, we first derive a result on the *sum of overlaps* in Lem. 11.

Interestingly, $S_{{{\,\textrm{Cl}\,}}}$ is *not* close to $P_{{{\,\textrm{Cl}\,}}}$ in diamond norm (see. Ch. 15 in Ref. [64]). To derive our main result, we instead construct an orthogonalized basis from the $Q_T^{\otimes n}$. Some properties of the orthogonalized basis are proven in Lem. 4, which also makes use of Lem. 11.

#### Lemma 11

(Overlap of stochastic Lagrangian sub-spaces). We have $\left( Q_T \big | Q_{T'} \right) \ge 0$ for all $T,T'\in \Sigma _{t,t}$. Moreover, for all $T\in \Sigma _{t,t}$ the sum of overlaps is

93 $$\begin{aligned} \sum _{T'\in \Sigma _{t,t}} \left( Q_T \big | Q_{T'} \right) ^n = (-2^{-n}; 2)_{t-1} \le 1+ t2^{t-n}, \end{aligned}$$

where $(-2^{-n}; 2)_{t-1} = \prod _{r=0}^{t-2}(1+2^{r-n})$ and the last inequality holds for $n + 2 \ge t + \log _2(t)$.

#### Proof

Denote by $\textrm{Stab}(n)$ the set of stabilizer states on *n* qubits. Since the operators *r*(*T*) are entry-wise non-negative, we have $\left( Q_T \big | Q_{T'} \right) =2^{-t}{{\,\textrm{Tr}\,}}(r(T)^\dagger r(T'))\ge 0$. Note that $r(T)^\dagger = r({\tilde{T}})$ for a suitable $\tilde{T}\in \Sigma _{t,t}$ (cp. Thm. 4). We obtain

94 $$\begin{aligned} \sum _{T'\in \Sigma _{t,t}} \left( Q_T \big | Q_{T'} \right) ^n&=\frac{1}{2^{tn}}\sum _{T'\in \Sigma _{t,t}}{{\,\textrm{Tr}\,}}\left[ r({\tilde{T}})^{\otimes n} r(T')^{\otimes n}\right] \nonumber \\&{\mathop {=}\limits ^{\dagger }}\frac{2^n\prod _{r=0}^{t-2}(2^r+2^n)}{2^{tn}}{{\,\textrm{Tr}\,}}\left[ r({\tilde{T}})^{\otimes n}{\mathbb {E}}_{s\in \mathrm {Stab(n)}}(\left. \left| {s}\right. \right\rangle \!\! \left. \left\langle {s}\right. \right| ^{\otimes t})\right] \nonumber \\&=\frac{2^n\prod _{r=0}^{t-2}(2^r+2^n)}{2^{tn}}{\mathbb {E}}_{s\in \mathrm {Stab(n)}}\left\langle s^{\otimes t} \right| r({\tilde{T}})^{\otimes n} \left| s^{\otimes t} \right\rangle \nonumber \\&{\mathop {=}\limits ^{\ddagger }}\frac{2^n\prod _{r=0}^{t-2}(2^r+2^n)}{2^{tn}}\nonumber \\&=\prod _{r=0}^{t-2}(1+2^{r-n}) \nonumber \\&\le \left( 1+2^{t-2-n}\right) ^{t-1} \nonumber \\&{\mathop {\le }\limits ^{*}} \exp \left( (t-1) 2^{t-n-2}\right) , \end{aligned}$$

where we have again used [42, Thm. 5.3] in $\dagger$ and in $\ddagger$ that $\left\langle s^{\otimes t} \right| r(T)^{\otimes n} \left| s^{\otimes t} \right\rangle =1$ for all $T\in \Sigma _{t,t}$ and all $s\in \textrm{Stab}(n)$ (compare Ref. [42, Eq. (4.10)]). Finally, in $*$ we have used the “inverse Bernoulli inequality” $(1+x)^r \le e^{rx}$ which holds for all $x\in {\mathbb {R}}$ and $r\ge 0$. By assumption, the following holds

95 $$\begin{aligned} 0 \ge t + \log _2(t) - n - 2 \quad \Rightarrow \quad 1 \ge t 2^{t-n-2} \ge (t-1)2^{t-n-2}. \end{aligned}$$

Thus, we can use the inequality $e^x \le 1+2x$ for $0\le x\le 1$ to obtain

96 $$\begin{aligned} \sum _{T'\in \Sigma _{t,t}} \left( Q_T \big | Q_{T'} \right) ^n&\le 1 + (t-1) 2^{t-n-1} \nonumber \\&\le 1 + t 2^{t-n}. \end{aligned}$$

$$\square $$

#### Lemma 12

Let $S_{{{\,\textrm{Cl}\,}}}$ be the Clifford frame operator and $\Gamma$ the corresponding Gram matrix, i. e. $\Gamma _{T,T'}=\left( Q_T \big | Q_T' \right) ^{n}$. Then the following holds

97 $$\begin{aligned} \left\| S_{{{\,\textrm{Cl}\,}}}-P_{{{\,\textrm{Cl}\,}}} \right\| _\infty = \left\| \Gamma -\mathbbm {1} \right\| _\infty \le (-2^{-n};2)_{t-1} - 1 \le t 2^{t-n}, \end{aligned}$$

where $(-2^{-n}; 2)_{t-1} = \prod _{r=0}^{t-2}(1+2^{r-n})$ and the last inequality holds for $n + 2 \ge t + \log _2(t)$.

#### Proof

Define the *synthesis operator* of the frame as the map

98 $$\begin{aligned} V:\,{\mathbb {C}}^{|\Sigma _{t,t}|}\rightarrow {{\,\textrm{Cl}\,}}(n)', \quad V=\sum _{T\in \Sigma _{t,t}} \left. \left| {Q_T^{\otimes n}}\right. \right) \!\! \left. \left\langle {e_T}\right. \right| , \end{aligned}$$

where $e_T$ is the standard basis of the domain. Then, we have clearly $\Gamma =V^\dagger V$ and $S_{{{\,\textrm{Cl}\,}}}|_{{{\,\textrm{Cl}\,}}(n)'}=VV^\dagger$. Since $S_{{{\,\textrm{Cl}\,}}}$ and $P_{{{\,\textrm{Cl}\,}}}$ are both identically zero on $\left( {{\,\textrm{Cl}\,}}(n)'\right) ^{\perp }$, this part does not contribute to the spectral norm. From this it is clear that

99 $$\begin{aligned} \left\| S_{{{\,\textrm{Cl}\,}}}-P_{{{\,\textrm{Cl}\,}}} \right\| _\infty = \left\| \Gamma -\mathbbm {1} \right\| _\infty . \end{aligned}$$

Moreover, we can compute

100 $$\begin{aligned} \left\| \Gamma -\mathbbm {1} \right\| _\infty&= \left\| \sum _T \sum _{T,T'} \left( Q_T \big | Q_{T'} \right) ^n \left. \left| {e_T}\right. \right\rangle \!\! \left. \left\langle {e_{T'}}\right. \right| \right\| _\infty \nonumber \\&\le \max _{T}\sum _{T'\ne T} \left( Q_T \big | Q_{T'} \right) ^n \nonumber \\&= (-2^{-n};2)_{t-1} - 1, \end{aligned}$$

where we have used that the spectral norm of Hermitian operators is bounded by the max-column norm and inserted the exact result of Lemma 11 in the last step. Finally, said lemma provides the desired bound for $n+2\ge t+\log _2 t$. $\square$

### Proof of Lemmas for Theorem 1

#### Lemma 2

(Overlap bound). Let *K* be a single qubit gate which is not contained in the Clifford group. Then, there is a constant $c(K)>0$ such that

26 $$\begin{aligned} \eta _{K,t}:= \max _{\begin{array}{c} T\in \Sigma _{t,t}-S_t\\ T'\in \Sigma _{t,t} \end{array}} \frac{1}{3} \left| \left( Q_T \right| {{\,\textrm{Ad}\,}}_K^{\otimes t}+{{\,\textrm{Ad}\,}}_{K^{\dagger }}^{\otimes t}+\textrm{id} \left| Q_{T'} \right) \right| \le 1-c(K)\log ^{-2}(t). \end{aligned}$$

The proof of Lemma 2 is based on two results. The first states that the basis elements *r*(*T*) of the commutant of tensor powers of the Clifford group either belong to the commutant of the powers of the unitary group, or else are far away from it.

#### Lemma 13

(Haar symmetrization). For all *t* and for all $T\in \Sigma _{t,t}\setminus S_t$, it holds that

101 $$\begin{aligned} \left( Q_T \right| P_{\textrm{H}} \left| Q_T \right) =2^{-t} \left\| P_\textrm{H}[r(T)] \right\| _{2}^2 \,\le \, \frac{7}{8}, \end{aligned}$$

where $Q_T$ is as in Eq. (21) and $P_{\textrm{H}}=\textrm{M}_t(\mu _\textrm{H})$ is the *t*-th moment operator of the single-qubit unitary group ${{\,\textrm{U}\,}}(2)$.

The proof is given in Sect. 6.3. In Appendix C, we show that the constant 7/8 cannot be improved below 7/10, by exhibiting a *T* that attains this bound.

The second ingredient to Lemma 2 is a powerful theorem by Varjú [53]. Here, we specialize this theorem to the unitary group:

#### Theorem 5

([53, Thm. 6]. Let $\nu$ be a probability measure on ${{\,\textrm{U}\,}}(d)$. Consider the averaging operator $T_{v}(\nu )$ on a irreducible representation $\pi _{v}:\,{{\,\textrm{U}\,}}(d)\rightarrow \textrm{End}(W_v)$ parameterized by highest weight $v\in {\mathbb {Z}}^d$:

102 $$\begin{aligned} T_v(\nu ) :=\int _{{{\,\textrm{U}\,}}(d)} \pi _v(U) \, \textrm{d}\nu (U). \end{aligned}$$

Then there are numbers $C(d)>0$ and $r_0>0$ such that

103 $$\begin{aligned} \Delta _r(\nu ):=1-\max _{0<|v|\le r}\left\| T_v(\nu ) \right\| _\infty \ge C(d)\Delta _{r_0}(\nu )\log ^{-2}(r), \end{aligned}$$

where $$|v|^2=\sum _{i}v_i^2$$.

#### Proof of Lemma 2

Consider the probability measure $\xi _K$ that draws uniformly from the set $\{K, K^{\dagger }, \mathbbm {1}\}$. Moreover, define $\nu _K$ on ${{\,\textrm{U}\,}}(2)$ as the average of the uniform measure on $\{H, S, S^3\}$ and $\xi _K*\xi _K$. Hence, the according moment operator is

104 $$\begin{aligned} \textrm{M}_t(\nu _K):=&\frac{1}{6} ({{\,\textrm{Ad}\,}}_H^{\otimes t}+{{\,\textrm{Ad}\,}}_S^{\otimes t}+({{\,\textrm{Ad}\,}}_S^3)^{\otimes t})+\frac{1}{2} \textrm{M}_{t}(\xi _K*\xi _K)\nonumber \\ =&\frac{1}{6} ({{\,\textrm{Ad}\,}}_H^{\otimes t}+{{\,\textrm{Ad}\,}}_S^{\otimes t}+({{\,\textrm{Ad}\,}}_S^3)^{\otimes t})+\frac{1}{2} \textrm{M}_t(\xi _K)^2. \end{aligned}$$

As the Clifford group augmented with any non-Clifford gate is universal [65, Thm. 6.5], so is the probability measure $\nu _K$.

It follows from the representation theory of the unitary group (see App. B) that the representation $U\mapsto {{\,\textrm{Ad}\,}}_U^{\otimes t}$ does not contain irreducible representations $W_v$ with highest weight of length $|v|>\sqrt{2}t$. Thus, we can decompose into these irreducible representations as follows:

105 $$\begin{aligned} \left\| \textrm{M}_t(\nu _K) - P_{\textrm{H}} \right\| _\infty&= \left\| \bigoplus _{|v|\le \sqrt{2}t} \left( T_v(\nu _K) - T_v(\mu _\textrm{H})\right) \otimes \textrm{id}_{m_v} \right\| _\infty \nonumber \\&\le \left\| \bigoplus _{0<|v|\le \sqrt{2}t} T_v(\nu _K) \right\| _\infty \nonumber \\&= \max _{0<|v|\le \sqrt{2}t} \left\| T_v(\nu _K) \right\| _\infty \nonumber \\&= 1 - \Delta _{\sqrt{2}t}(\nu _K). \end{aligned}$$

Here, $m_v$ denotes the multiplicity of the irreducible representation $W_v$ (possibly zero). In the second step we have used that $P_{\textrm{H}}$ has only support on the trivial irreducible representation $v=0$, where both $P_{\textrm{H}}$ and $\textrm{M}_t(\nu _K)$ act as identity and thus cancel. Hence, only non-trivial irreducible representations are contributing. To bound $\Delta _{\sqrt{2}t}(\nu _K)$, we can invoke Theorem 5 combined with the fact that for any universal probability measure the restricted gap is non-zero: $\Delta _{r}(\nu _K)>0$ for all $r\ge 1$ (compare e.g. Ref. [27]). Hence, we obtain

106 $$\begin{aligned} \Delta _{\sqrt{2}t}(\nu _K)\ge & {} C(2)\Delta _{r_0}(\nu _K)\log ^{-2}\left( \sqrt{2}t\right) \nonumber \\\ge & {} \frac{1}{4}C(2)\Delta _{r_0}(\nu _K)\log ^{-2}(t)=:c'(K)\log ^{-2}(t) > 0, \end{aligned}$$

where $c(K)>0$. Therefore, we have

107 $$\begin{aligned} \left\| \textrm{M}_t(\nu _K) - P_{\textrm{H}} \right\| _\infty \le 1-\Delta _{\sqrt{2}t}(\nu _K)\le 1-c'(K)\log ^{-2}(t)=:\kappa _{t,K}, \end{aligned}$$

Furthermore, consider the operator

108 $$\begin{aligned} X_T:=\frac{(\textrm{id}-P_{\textrm{H}})Q_T}{\left\| (\textrm{id}-P_{\textrm{H}})Q_T \right\| _2}. \end{aligned}$$

We obtain

109 $$\begin{aligned} \left\| \textrm{M}_t(\nu _K) - P_{\textrm{H}} \right\| _\infty&= \max _{\Vert X\Vert _2=1}\left| \left( X \right| \textrm{M}_t(\nu _K) -P_{\textrm{H}} \left| X \right) \right| \nonumber \\&\ge \frac{\left| \left( X_T \right| \textrm{M}_t(\nu _K) -P_{\textrm{H}} \left| X_T \right) \right| }{\Vert X_T\Vert ^2_2}\nonumber \\&=\frac{\left| \left( Q_T \right| (\textrm{id}-P_{\textrm{H}})\textrm{M}_t(\nu _K) (\textrm{id}-P_{\textrm{H}}) \left| Q_T \right) \right| }{\left( Q_T \right| (\textrm{id}-P_{\textrm{H}})^2 \left| Q_T \right) }\nonumber \\&=\frac{|\left( Q_T \right| \textrm{M}_t(\nu _K) \left| Q_T \right) - \left( Q_T \right| P_{\textrm{H}} \left| Q_T \right) |}{1-\left( Q_T \right| P_{\textrm{H}} \left| Q_T \right) } \nonumber \\&\ge \frac{\left( Q_T \right| \textrm{M}_t(\nu _K) \left| Q_T \right) - \left( Q_T \right| P_{\textrm{H}} \left| Q_T \right) }{1-\left( Q_T \right| P_{\textrm{H}} \left| Q_T \right) }. \end{aligned}$$

In the fourth step, we again used the properties of the Haar projector as in Eq. (79). Combining this with (107) and Lemma 13 we obtain

110 $$\begin{aligned} \left( Q_T \right| \textrm{M}_t(\nu _K) \left| Q_T \right) \le \kappa _{t,K}+(1-\kappa _{t,K})\left( Q_T \right| P_{\textrm{H}} \left| Q_T \right) \le 1-\frac{1}{8}c'(K)\log ^{-2}(t).\nonumber \\ \end{aligned}$$

We can use that $\left( Q_T \right| {{\,\textrm{Ad}\,}}_S^{\otimes t} \left| Q_T \right) =\left( Q_T \right| {{\,\textrm{Ad}\,}}_{S^3}^{\otimes t} \left| Q_T \right) =\left( Q_T \right| {{\,\textrm{Ad}\,}}_H^{\otimes t} \left| Q_T \right) =1$ for all $T\in \Sigma _{t,t}$ because $Q_T=2^{-t/2}r(T)$ commutes with the *t*-th diagonal action of the single-qubit Clifford group (compare [42, Lem. 4.5]). We immediately obtain

111 $$\begin{aligned} \left( Q_T \right| \textrm{M}_t(\xi _K)^2 \left| Q_T \right) \le 1-\frac{1}{4}c'(K)\log ^{-2}(t). \end{aligned}$$

From the Cauchy-Schwarz inequality, we now get

112 $$\begin{aligned} \left| \left( Q_T \right| \textrm{M}_t(\xi _K) \left| Q_{T'} \right) \right|&\le \sqrt{\left( Q_T \right| \textrm{M}_t(\xi _K)^2 \left| Q_T \right) }\nonumber \\&\le \sqrt{1-\frac{1}{4}c'(K)\log ^{-2}(t)}\nonumber \\&\le 1-\frac{1}{8} c'(K)\log ^{-2}(t)\nonumber \\&=: 1- c(K)\log ^{-2}(t), \end{aligned}$$

where we have used that $c'(K)\log ^{-2}(t) \le \Delta _{\sqrt{2}t}(\nu _K)\le 1$ such that we can use the inequality $\sqrt{1-x}\le 1-x/2$ for $x\le 1$. This shows the claimed statement. $\square$

#### Remark 2

(*Quantum gates with algebraic entries*). If we restrict to gates *K* that have only algebraic entries, we can apply the result from Ref. [66] and save the additional overhead of $\log ^2(t)$ in the scaling. This applies to the *T*-gate and for essentially all gates that might be used in practical implementations. Here, we have chosen the more general approach.

#### Remark 3

(*Implications for quantum information processing*). Theorem 5 has miscellaneous implications for quantum information processing. E.g. we can immediately combine this bound with the local-to-global lemma in Ref. [23, Lem. 16] to extend Ref. [24, Cor. 7] to gate sets with non-algebraic entries at the cost of an additional overhead of $\log ^2(t)$ in the scaling. The bottleneck to loosen the invertibility assumption as well is the local-to-global lemma which only works for Hermitian moment operators (symmetric distributions). Work to lessen the assumption of invertibility has been done in Ref. [67]. Extending this would be an interesting application which we, however, do not pursue in this work.

#### Lemma 4

(Properties of the constructed basis). Let $\{T_j\}_{j=1}^{|\Sigma _{t,t}|}$ be an enumeration of the elements of $\Sigma _{t,t}$ such that the first *t*! spaces $T_j$ correspond to the elements of $S_t$. Then, the $\{E_j\}$ constitutes an orthogonal (but not normalized) basis, where

30 $$\begin{aligned} E_j :=\sum _{i=1}^jA_{i,j}\,Q_{T_i}^{\otimes n}:= \sum _{i=1}^j\left[ \sum _{\begin{array}{c} \Pi \in S_j\\ \Pi (j)=i \end{array}}{{\,\textrm{sign}\,}}(\Pi )\prod _{l=1}^{j-1} \left( Q_{T_l} \big | Q_{T_{\Pi (l)}} \right) ^n\right] \,Q_{T_i}^{\otimes n}. \end{aligned}$$

Denote by $N_i$ the defect space of $T_i$. For $n\ge \frac{1}{2}(t^2+5t)$, we have

31 $$\begin{aligned} |A_{i,j}|&\le 2^{t^3+4t^2+6t-n|\dim N_i-\dim N_j|}, \qquad \forall i,j, \end{aligned}$$

32 $$\begin{aligned} |A_{i,j}|&\le 2^{2t^2+10t-n}, \qquad \forall i\ne j. \end{aligned}$$

Moreover, it holds that

33 $$\begin{aligned} 1-2^{t^2+7t-n}\le A_{j,j}\le 1+2^{t^2+7t-n}. \end{aligned}$$

#### Proof

The form of (30) is up to a constant the determinant formulation of the Gram-Schmidt procedure. First, note that the number of permutations of *n* elements with no fixed points is known from Ref. [68] to be

113 $$\begin{aligned} D(n)=n!\sum _{r=0}^n\frac{(-1)^r}{r!}\le 2\frac{n!}{e} \end{aligned}$$

for $n\ge 1$. Here, *D* stands for “derangement” as permutations without fixed points are sometimes called. Then, the number of permutations having exactly *k* fixed points is ${n\atopwithdelims ()k}$ many choices of *k* points times the number $D(n-k)$ of deranged permutations on the remaining $n-k$ objects:

114 $$\begin{aligned} p(n,k):={n\atopwithdelims ()k}D(n-k)\le 2e^{-1}\frac{n!}{k!}. \end{aligned}$$

The following estimate for certain sums involving *p*(*n*, *k*) will shortly become useful. Note that we have for any $M,L\in {\mathbb {N}}$ and $m\in {\mathbb {R}}$ such that $2^m>M-L$ and $M\ge L\ge 1$:

115 $$\begin{aligned} \sum _{k=0}^{M-L} p(M,k) 2^{-m(M-k)}\le & {} \frac{2}{e} \sum _{k=0}^{M-L} 2^{-mM} M! \frac{2^{mk}}{k!} \nonumber \\\le & {} \frac{2}{e} 2^{-mM} (M-L+1) M!\frac{2^{m(M-L)}}{(M-L)!} \nonumber \\\le & {} M^{L+1} 2^{-mL}. \end{aligned}$$

Here, we have used in the second inequality that $2^{mk}/k!$ is monotonically increasing for $k\le M-L < 2^m$ and a standard bound on binomial coefficients in the last step.

We start by bounding the diagonal coefficients $A_{j,j}$. The idea is to divide the set of permutations into sets of permutations with exactly *k* fixed points. For any such permutation, the product of overlaps collapses to only $j-1-k$ non-trivial inner products. By assumption $n\ge \frac{1}{2}(t^2+5t)\ge t+\log _2 t$, thus we can be bound any of those using Lemma 11 as

116 $$\begin{aligned} \left( Q_T \big | Q_{T'} \right) ^n \le t2^{t-n}, \quad \text { for all } T\ne T'. \end{aligned}$$

Note that the trivial permutation (corresponding to $k=j-1$ fixed points) contributes by exactly 1 to the sum. Thus, we find the following bound using Eq. (115) with $M=j-1$, $L=1$ and $m=n-t-\log _2 t$:

117 $$\begin{aligned} A_{j,j}&=|A_{j,j}| \le \sum _{\pi \in S_{j-1}} \prod _{l=1}^{j-1} \left( Q_l \big | Q_{\pi (l)} \right) ^n \nonumber \\&\le 1 + \sum _{k=0}^{j-2} p(j-1,k) 2^{-(n-t-\log _2 t)(j-1-k)} \nonumber \\&\le 1 + (j-1)^2 \, 2^{-n+t+\log _2 t} \nonumber \\&< 1 + 2^{t^2 + 7t - n}, \end{aligned}$$

where we have used Eq. (15) in the last step as $j-1 < j \le |\Sigma _{t,t}|\le 2^{\frac{1}{2} (t^2+5t)}$. Using the reverse triangle inequality, we get a lower bound in the same way:

118 $$\begin{aligned} A_{j,j}=|A_{j,j}| \ge 1 - \left| \sum _{\pi \in S_{j-1}\setminus \textrm{id}}{{\,\textrm{sign}\,}}(\pi ) \prod _{l=1}^{j-1} \left( Q_l \big | Q_{\pi (l)} \right) ^n\right| \ge 1 - 2^{t^2 + 7t - n}. \end{aligned}$$

Next, we will bound the off-diagonal terms $A_{i,j}$. It is well known that every permutation $\Pi \in S_j$ can be written as a product of disjoint cycles. Given a $\Pi \in S_j$ with $\Pi (j)=i$, consider the cycle $j\mapsto i\mapsto i_1\mapsto i_2\mapsto \dots i_r\mapsto j$ in $\Pi$. Then, we have the bound

119 $$\begin{aligned} \prod _{l=1}^{j-1}\left( Q_{T_l} \big | Q_{T_{\Pi (l)}} \right) ^n&\le \left( Q_{T_i} \big | Q_{T_{i_1}} \right) ^n \dots \left( Q_{T_{i_r}} \big | Q_{T_j} \right) ^n\nonumber \\&\le 2^{-n(|\dim N_i-\dim N_{i_1}|+\dots |\dim N_{i_r}-\dim N_{j}|)}\nonumber \\&\le 2^{-n|\dim N_i-\dim N_{j}|}, \end{aligned}$$

where we have used Lemma 3, the triangle inequality and a telescope sum. We set $L:=|\dim N_i-\dim N_{j}|$ and split the sum over permutations into those with more than or equal to $j-L$ many fixed points and those with less. In the first case, we use Eq. (119) to bound the overlaps, in the second case we use Eq. (115) as before. This yields the following bound

120 $$\begin{aligned} |A_{i,j}|&\le \sum _{\begin{array}{c} \Pi \in S_j\\ \Pi (j)=i \end{array}}\prod _{l=1}^{j-1}\left( Q_{T_l} \big | Q_{T_{\Pi (l)}} \right) ^n\nonumber \\&\le \sum _{k=j-L}^{j-1} p(j,k) 2^{-nL} + \sum _{k=0}^{j-L-1} p(j,k) 2^{-(n-t-\log _2t)(j-1-k)}\nonumber \\&\le \frac{2}{e} \sum _{k=j-L}^{j-1}\frac{j!}{k!} 2^{-nL} + 2^{n-t-\log _2t} j^{L+2} \, 2^{-(n-t-\log _2t)(L+1)} \nonumber \\&\le L \frac{j!}{(j-L)!} 2^{-nL} + j^{L+2} \, 2^{-(n-t-\log _2t)L} \nonumber \\&\le L j^L 2^{-nL} + j^{L+2} \, 2^{-(n-t-\log _2t)L} \nonumber \\&\le L |\Sigma _{t,t}|^{L+2} \, 2^{-(n-t-\log _2t)L} \nonumber \\&\le 2^{\log _2 L }2^{\frac{1}{2} (t^2+5t)(L+2)} \, 2^{(t +\log _2t-n)L} \nonumber \\&= 2^{t^2+5t} 2^{(\frac{1}{2} t^2 + \frac{5}{2} t + t +\log _2t-n)L} \nonumber \\&\le 2^{\frac{1}{4} t^3 + \frac{11}{4} t^2 + 5t + (\frac{t}{2} + 1)\log _2 t - nL} \nonumber \\&\le 2^{t^3 + 4 t^2 + 6t - n|\dim N_i-\dim N_j|}, \end{aligned}$$

where we have used again $j\le |\Sigma _{t,t}|$ and $L\le t/2$.

Note that we can alternatively bound $A_{i,j}$ for $i \ne j$ using that the identity is not an allowed permutation, i. e. only permutations with less than $j-2$ fixed points can appear. With Eqs. (115) and (116), we get the following inequality

121 $$\begin{aligned} |A_{i,j}|&\le \sum _{k=0}^{j-2} p(j,k) 2^{-(n-t-\log _2t)(j-1-k)}\nonumber \\&\le j^3 2^{-(n-t-\log _2t)} \nonumber \\&\le 2^{\frac{3}{2} t^2 + \frac{15}{2} t + t + \log _2 t - n} \nonumber \\&\le 2^{2t^2 + 10 t - n}. \end{aligned}$$

$$\square $$

### Proof of Haar symmetrization Lemma 13

#### Lemma 13

(Haar symmetrization). For all *t* and for all $T\in \Sigma _{t,t}\setminus S_t$, it holds that

101 $$\begin{aligned} \left( Q_T \right| P_{\textrm{H}} \left| Q_T \right) =2^{-t} \left\| P_\textrm{H}[r(T)] \right\| _{2}^2 \,\le \, \frac{7}{8}, \end{aligned}$$

where $Q_T$ is as in Eq. (21) and $P_{\textrm{H}}=\textrm{M}_t(\mu _\textrm{H})$ is the *t*-th moment operator of the single-qubit unitary group ${{\,\textrm{U}\,}}(2)$.

For an analysis of the tightness of the bound, see “Appendix C”. Recall that

122 $$\begin{aligned} P_\textrm{H}[A] := \int _{U(2)} U^{\otimes t} A (U^\dagger )^{\otimes t} {\textrm{d}}\mu _\textrm{H}(U). \end{aligned}$$

Let $P_D$ be the Haar averaging operator, restricted to the diagonal unitaries. As it averages over a subgroup, $P_D$ is a projection with range a super-set of $P_\textrm{H}$. By applying $P_D$ to *r*(*T*), we can turn the statement (101) from one involving *Hilbert space* geometry to one about the *discrete* geometry of stochastic Lagrangians. Indeed,

$$\begin{aligned} 2^{-t} \left\| P_\textrm{H}[r(T)] \right\| _{2}^2&= 2^{-t} \left\| P_\textrm{H}[P_D[r(T)]] \right\| _{2}^2 \\&\le 2^{-t} \left\| P_D[r(T)] \right\| _{2}^2 \\&= 2^{-t} \Big (r(T), P_D[r(T)]\Big ) \\&= 2^{-t} \sum _{(x,y)\in T} \sum _{(x',y')\in T} \big ( \left. \left| {x}\right. \right\rangle \!\! \left. \left\langle {y}\right. \right| , P_D[\left. \left| {x'}\right. \right\rangle \!\! \left. \left\langle {y'}\right. \right| ] \big ) \\&= 2^{-t} \sum _{(x,y)\in T} \sum _{(x',y')\in T} \big ( \left. \left| {x}\right. \right\rangle \!\! \left. \left\langle {y}\right. \right| , \int _0^{2\pi } e^{i 2\phi (h(x') - h(y'))} \left. \left| {x'}\right. \right\rangle \!\! \left. \left\langle {y'}\right. \right| \textrm{d}\,\phi \big ) \\&= 2^{-t} |\{ (x,y) \in T \,|\, h(x)=h(y) \}| \\&={\text {Pr}}_{(x,y)}[ h(x) = h(y) ], \end{aligned}$$

i.e., the overlap is upper-bounded by the probability that a uniformly sampled element (*x*, *y*) of *T* has components of equal Hamming weight.

We will bound the probability in slightly different ways for spaces *T* with trivial (i.e., zero-dimensional) and non-trivial defect spaces.

*a. Case I: trivial defect sub-spaces* In this case, $T=\{ (Oy, y) \,|\, y\in {\mathbb {F}}_2^t\}$ for some orthogonal stochastic matrix *O*. The next proposition treats a slightly more general situation.

#### Proposition 4

(Hamming bound). Let $O\in \textrm{GL}({\mathbb {F}}_2^t)$. Assume *O* has a column of Hamming weight *r*. Then the probability that *O* preserves the Hamming weight of a vector *y* chosen uniformly at random from ${\mathbb {F}}_2^t$ satisfies the bound

123 $$\begin{aligned} \textrm{Pr}_y[ h(Oy) = h(y) ] \le \frac{1}{2} + \left\{ \begin{array}{ll} 2^{-(r+1)} {{r+1}\atopwithdelims (){(r+1)/2}}\quad &{}r \text { odd} \\ 0&{}r\text { even}. \end{array} \right. \end{aligned}$$

The bound in Eq. (123) decreases monotonically in *r*. Orthogonal stochastic matrices *O* satisfy $r=1\mod 4$, so the smallest non-trivial *r* that can appear is $r=5$, for which the bound gives .81.

The proof idea is as follows: For each $y\in {\mathbb {F}}_2^t$, the two vectors $y, y+e_1$ differ in Hamming weight by $\pm 1$. But, if $h(e_1)\ne 1$, then $h(Oy)-h(O(y+e_1))$ tends not to be $\pm 1$. In such cases, *O* does not preserve weights for *both* *y* and $y+e_1$. Applying this observation to randomly chosen vectors, we can show the existence of many vectors for which *O* changes the Hamming weight.

#### Proof of Proposition 4

Assume without loss of generality that the first *r* entries of $O e_1$ are 1, and the remaing $t-r$ entries are 0.

Let *y* be a uniformly distributed random vector on ${\mathbb {F}}_2^t$, notice that also *Oy*, and $O(y+e_1)$ are uniformly distributed. Using the union bound, we find that

$$\begin{aligned} \textrm{Pr}[h(Oy)=h(y)]&= 1- \textrm{Pr}[h(Oy)\ne h(y)] \\&= 1- \frac{1}{2}\big ( \textrm{Pr}[h(Oy)\ne h(y)] + \textrm{Pr}[h(Oy+Oe_1)\ne h(y+e_1) ] \big )\\&\le 1- \frac{1}{2} \textrm{Pr}[h(Oy)\ne h(y) \,\vee \, h(Oy+Oe_1)\ne h(y+e_1) ]\\&= \frac{1}{2} + \frac{1}{2} \textrm{Pr}[h(Oy)= h(y) \,\wedge \, h(Oy+Oe_1)= h(y+e_1) ]\\&\le \frac{1}{2} + \frac{1}{2} \textrm{Pr}[ h(Oy)-h(Oy+Oe_1) = \pm 1]. \end{aligned}$$

We would like to compute $\textrm{Pr}[ h(Oy)-h(O(y+e_1)) = \pm 1]$. The vector $O(y+e_1)=O(y)+O(e_1)$ arises from *O*(*y*) by flipping the first *r* components. This operation changes the Hamming weight by $\pm 1$ if and only if the number of ones in the first *r* components of *O*(*y*) equals $(r\pm 1)/2$. For even *r*, this condition cannot be met, and correspondingly $\textrm{Pr}[ h(Oy)-h(O(y+e_1)) = \pm 1]=0$.

In case of odd *r*, this probability becomes

124 $$\begin{aligned} \textrm{Pr}[ h(Oy)-h(O(y+e_1))&= \pm 1]= 2^{-r} {{r}\atopwithdelims (){(r-1)/2}} + 2^{-r} {{r}\atopwithdelims (){(r+1)/2}}\nonumber \\&= 2^{-r} {{r+1}\atopwithdelims (){(r+1)/2}}. \end{aligned}$$

$$\square $$

*b. Case II: non-trivial defect sub-spaces* We now turn to Lagrangians *T* with a non-trivial defect subspace.

#### Proposition 5

(Defect Hamming bound). Let $\{0\}\ne N\subset {\mathbb {F}}_2^t$ be isotropic. There exists an $n\in N$ such that if *x* is chosen uniformly at random from $N^\perp$, then

$$\begin{aligned} \textrm{Pr}_{x\in N^\perp }[ h(x) = h(x+n) ] \le \frac{3}{4}. \end{aligned}$$

What is more, let *T* be a stochastic Lagrangian with non-trivial defect sub-spaces. Then, for an element (*x*, *y*) drawn uniformly from *T*, we have

$$\begin{aligned} {\text {Pr}}_{(x,y)\in T}[ h(x) = h(y) ] \le \frac{7}{8}. \end{aligned}$$

#### Proof

Let $d=\dim N$. Consider a $t \times d$ column-generator matrix $\Gamma$ for *N*. Permuting coordinates of ${\mathbb {F}}_2^t$ and adopting a suitable basis, there is no loss of generality in assuming that $\Gamma$ is of the form

$$\begin{aligned} \Gamma = \begin{pmatrix} G \\ \mathbbm {1}_{d} \end{pmatrix}, \qquad G \in {\mathbb {F}}_2^{(t-d)\times d}. \end{aligned}$$

Note that

$$\begin{aligned} \gamma = \begin{pmatrix} \mathbbm {1}_{t-d},&G \end{pmatrix} \end{aligned}$$

is a row-generator matrix for $N^\perp$. Indeed, the row-span has dimenion $t-d$ and the matrices fulfill

$$\begin{aligned} \gamma \Gamma = G + G = 0, \end{aligned}$$

i.e., the inner product between any column of $\Gamma$ and any row of $\gamma$ vanishes. It follows that elements $n\in N$, $x\in N^\perp$ are exactly the vectors of respective form

$$\begin{aligned} n=(\,\underbrace{G \tilde{n}}_{t-d},\, \underbrace{\tilde{n}}_d\,),\; \tilde{n}\in {\mathbb {F}}_2^d; \qquad x=(\,\underbrace{\tilde{x}}_{t-d},\, \underbrace{G^T \tilde{x}}_d\,),\; \tilde{x}\in {\mathbb {F}}_2^{t-d}. \end{aligned}$$

In particular, if *x* is drawn uniformly from $N^\perp$, then the first $t-d$ components are uniformly distributed in ${\mathbb {F}}_2^{t-d}$. For now, we restrict to the case where *G* has a column, say the first, with $r\ne 1$ non-zero entries. We then choose $n=(Ge_1, e_1)$ and argue as in Eq. (124) to obtain

125 $$\begin{aligned} \textrm{Pr}_{x\in N^\perp }[ h(x) = h(x+n)] \le \sup _{1\ne r\text { odd}} 2^{-r} {{r+1}\atopwithdelims (){(r+1)/2}} =\frac{3}{4} \qquad \text {(attained for } r=3).\nonumber \\ \end{aligned}$$

We are left with the case where all columns of *G* have Hamming weight 1. (If *N* is a defect subspace, then Def. 6.1 implies that every column of $\Gamma$ has Hamming weight at least 4. We treat the present case merely for completeness). As *N* is isotropic, the columns of $\Gamma$ have mutual inner product equal to 0:

$$\begin{aligned} \Gamma ^T \Gamma = 0 \qquad \Leftrightarrow \qquad G^TG=-\mathbbm {1}=\mathbbm {1}\mod 2. \end{aligned}$$

It follows that all columns have to be mutually orthogonal standard basis vectors $e_i\in {\mathbb {F}}_2^{t-d}$. Thus, by permutating the first $t-d$ coordinates of ${\mathbb {F}}_2^t$, we can assume that *G* is of the form

$$\begin{aligned} G = \begin{pmatrix} \mathbbm {1}_d \\ 0 \end{pmatrix}, \quad \Rightarrow \quad N =\{ (\tilde{n}\oplus 0_{t-2d}, \tilde{n}) \,|\, \tilde{n}\in {\mathbb {F}}_2^{d}\}, \quad N^\perp =\{ (\tilde{x}, \tilde{x}|_d) \,|\, \tilde{x}\in {\mathbb {F}}_2^{t-d}\}, \end{aligned}$$

where ${\tilde{x}}|_d$ denotes the restriction of ${\tilde{x}}$ to the first *d* components. Adding $n:=(e_1\oplus 0, e_1)$ to $x=(\tilde{x}, \tilde{x}|_d)$, the Hamming weight of the two parts change both by $\pm 1$, giving $h(x+n)=h(x)\pm 2$. Thus, we have $\textrm{Pr}[ h(x) = h(x+n)]=0$.

We have proven the first advertised claim. It implies the second one, as argued next. Let *N* be the left defect subspace of *T*. By Ref. [42, Prop. 4.17], we find the following.

- The restriction $\{x \,|\, (x,y) \in T \text { for some }y\}$ equals $N^\perp$.
- The stochastic Lagrangian *T* contains $N\oplus 0$.

Assume that (*x*, *y*) is distributed uniformly in *T*. By the first cited fact, *x* is distributed uniformly in $N^\perp$. By the second fact, $(x+n,y)$ follows the same distribution as (*x*, *y*), for each $n\in N$. Thus, repeating the argument in the proof of Proposition 4, we find that for any fixed $n\in N$:

$$\begin{aligned} \textrm{Pr}[ h(x) = h(y)]&= 1- \textrm{Pr}[h(x)\ne h(y)] \\&\le 1- \frac{1}{2} \textrm{Pr}[h(x)\ne h(y) \,\vee \, h(x+n)\ne h(y) ] \\&\le \frac{1}{2} + \frac{1}{2} \textrm{Pr}[ h(x)=h(x+n) ] \le \frac{7}{8}. \end{aligned}$$

$$\square $$

### Proof of Lemmas for Theorem 2

#### Lemma 5

(Relative $\varepsilon 2^{2tn}$-approximate Clifford *t*-designs). Suppose that $0 \le \varepsilon < 1$ is such that $g_{\textrm{Cl}}(\nu ,t)\le \varepsilon$. Then, $\nu$ is a relative $\varepsilon 2^{2tn}$-approximate Clifford *t*-design.

#### Proof

This follows similar to Ref. [24, Lem. 4& Lem. 30]. Denote by $|\Omega _{2^n}\rangle$ the maximally entangled state vector on ${\mathbb {C}}^{2^n}\otimes {\mathbb {C}}^{2^n}$. The condition in (5) is equivalent to

126 $$\begin{aligned} (1-\varepsilon )\rho _{\textrm{Cl}}\le \rho _{\nu }\le (1+\varepsilon )\rho _{\textrm{Cl}}, \end{aligned}$$

as an operator inequality, where

127 $$\begin{aligned} \rho _{\nu }:=(\Delta _{\nu }\otimes \mathbbm {1})(|\Omega _{2^n}\rangle \langle \Omega _{2^n}|)^{\otimes t}\qquad \text {and}\qquad \rho _{\textrm{Cl}}:=\rho _{\mu _{\textrm{Cl}}}. \end{aligned}$$

We have a decomposition of $({\mathbb {C}}^{2^n})^{\otimes t}$ into irreducible representations of the Clifford group:

128 $$\begin{aligned} ({\mathbb {C}}^{2^n})^{\otimes t}\cong \bigoplus _{\gamma }C_{\gamma }\otimes L_{\gamma }, \end{aligned}$$

where $\{C_\gamma \}$ is the set of all equivalence classes of irreducible representations of ${{\,\textrm{Cl}\,}}(n)$ that appear in the *t*-th order diagonal representation, and $L_\gamma$ are the corresponding multiplicity spaces (which by the double commutant theorem are irreducible representations of the commutant algebra –we have chosen *L* for Lagrangian). This implies that

129 $$\begin{aligned} |\Omega _{2^n}\rangle ^{\otimes t}\cong \sum _{\begin{array}{c} \gamma \end{array}}\sqrt{\frac{\dim L_\gamma \dim C_{\gamma }}{2^{nt}}} |\gamma ,\gamma \rangle \otimes |\Omega _{C_{\gamma }}\rangle \otimes |\Omega _{L_{\gamma }}\rangle , \end{aligned}$$

where $|\Omega _{L_{\gamma }}\rangle$ and $|\Omega _{C_{\gamma }}\rangle$ denote maximally entangling state vectors on two copies of $L_{\gamma }$ and $C_{\gamma }$, respectively. Indeed, observe that $\left. \left| {\Omega _{2^n}}\right. \right\rangle ^{\otimes t}=2^{-nt/2}{{\,\textrm{vec}\,}}(\mathbbm {1})$ and that the identity restricted to sub-spaces is just the identity on these sub-spaces. The prefactors then follow from normalizing the vectorized identity operators on the direct summands.

Since ${{\,\textrm{Cl}\,}}(n)$ acts via multiplication on the spaces $C_{\lambda }$, this implies that

130 $$\begin{aligned} \rho _{\textrm{Cl}}&=\int _{{{\,\textrm{Cl}\,}}(n)}(U\otimes \mathbbm {1})^{\otimes t}(|\Omega _{2^n}\rangle \langle \Omega _{2^n}|)^{\otimes t}(U^{\dagger }\otimes \mathbbm {1})^{\otimes t}{\textrm{d}\mu _{{{\,\textrm{Cl}\,}}}(U)}\nonumber \\&\cong \sum _{\gamma }\frac{\dim L_{\gamma }\dim C{\gamma }}{2^{nt}} (|\gamma \rangle \langle \gamma |)^{\otimes 2}\otimes \left( \frac{\mathbbm {1}_{C{\gamma }}}{\dim C{\gamma }}\right) ^{\otimes 2}\otimes |\Omega _{L_{\gamma }}\rangle \langle \Omega _{L_{\gamma }}|, \end{aligned}$$

where the second line follows from Schur’s lemma and the fact that $\int U^{\otimes t} \bullet (U^{\dagger })^{\otimes t}$ is trace preserving. The support of this operator is on the *symmetric subspace* $\vee ^t({\mathbb {C}}^{2^n}\otimes {\mathbb {C}}^{2^n})$ [24, Lem 30.1]. The minimal eigenvalue of this operator restricted to the symmetric subspace is

131 $$\begin{aligned} \min _{\gamma }\frac{\dim L_{\gamma }}{2^{nt}\dim C_{\gamma }}, \end{aligned}$$

which we now lower bound. Let $\gamma ^*$ denote the optimizer. By Schur-Weyl duality, the diagonal action of ${{\,\textrm{U}\,}}(2^n)$ on $({\mathbb {C}}^{2^n}\otimes {\mathbb {C}}^{2^n})^{\otimes t}$ decomposes as $\oplus _\lambda U_\lambda \otimes S_\lambda$ where as usual $U_\lambda$ are Weyl modules and $S_\lambda$ are Specht modules. Restricting this action to the Clifford group, the $U_\lambda$ further decompose into irreducible representations

$$\begin{aligned} U_\lambda \simeq \bigoplus _{\gamma \in I_\lambda } C_\gamma \otimes {\mathbb {C}}^{d_{\lambda ,\gamma }}, \end{aligned}$$

where $I_\lambda$ is the spectrum of $U_\lambda$ as a Clifford representation. Let $\Lambda _0$ be the set of all $\lambda$ such that $\gamma ^*\in I_\lambda$, then as a Clifford representation

132 $$\begin{aligned} ({\mathbb {C}}^{2^n}\otimes {\mathbb {C}}^{2^n})^{\otimes t} \simeq C_{\gamma ^*}\otimes \Big (\bigoplus _{\lambda \in \Lambda _0} S_\lambda \otimes {\mathbb {C}}^{d_{\lambda ,\gamma ^*}}\Big ) \oplus (\text {other irreducible representations}). \end{aligned}$$

Thus, as a vector space, we have

133 $$\begin{aligned} L_{\gamma ^*} = \bigoplus _{\lambda \in \Lambda _0} S_\lambda \otimes {\mathbb {C}}^{d_{\lambda ,\gamma ^*}}. \end{aligned}$$

In particular, for any $\lambda \in \Lambda _0$ we have that $\dim C_{\gamma ^*} \le \dim U_\lambda$ and $\dim L_{\gamma ^*} \ge \dim S_\lambda$. Thus we get the following bound for the minimal eigenvalue:

134 $$\begin{aligned} \frac{\dim L_{\gamma ^*}}{2^{nt}\dim C_{\gamma ^*}}\ge \min _{\lambda \in \textrm{Part}(t,2^n)}\frac{\dim S_{\lambda }}{2^{nt}\dim U_{\lambda }}\ge 2^{-2nt}. \end{aligned}$$

The rest of the proof follows as in Ref. [24, Lem. 4], mutatis mutandis. $\square$

In order to prove Lemma 8 we make use of the following result by [61] and Lemma 11 bounding certain sums of overlaps of the operators *r*(*T*).

#### Lemma 14

(Nachtergaele [61, Thm. 3]). Let $H_{[p,q]}$ for $[p,q]\subset [n]=\{1,\dots ,n\}$ be a family of positive semi-definite Hamiltonians with support on $({\mathbb {C}}^2)^{\otimes (q-p+1)}\subset ({\mathbb {C}}^2)^{\otimes n}$. Assume there is a constant $l\in {\mathbb {N}}$, such that the following conditions hold:

1. There is a constant $d_l>0$ for which the Hamiltonians satisfy
  135 $$\begin{aligned} 0\le \sum _{q=l}^n H_{[q-l+1,q]} \le d_l H_{[1,n]}. \end{aligned}$$
2. There are $Q_l\in {\mathbb {N}}$ and $\gamma _l>0$ such that there is a local spectral gap:
  136 $$\begin{aligned} \Delta \left( H_{[q-l+1,q]}\right) \ge \gamma _l, \quad \forall q \ge Q_l. \end{aligned}$$
3. Denote the ground state projector of $H_{[p,q]}$ by $G_{[p,q]}$. There exist $\varepsilon _l<1/\sqrt{l}$ such that
  137 $$\begin{aligned} \left\| G_{[q-l+2,q+1]}\left( G_{[1,q]}-G_{[1,q+1]}\right) \right\| _\infty \le \varepsilon _l, \quad \forall q \ge Q_l. \end{aligned}$$

Then, it holds that

138 $$\begin{aligned} \Delta \left( H_{[1,n]}\right) \ge \frac{\gamma _{l}}{d_{l}}\left( 1-\varepsilon _l\sqrt{l}\right) ^2. \end{aligned}$$

While conditions 1) and 2) are merely translation-invariance with finit range of interactions and frustration-freeness in disguise, the third condition is highly non-trivial and involves knowledge of the ground-space structure. Usually, finding the ground space in a basis can be just as hard as computing the spectral gap in the first place. Fortunately, the ground space structure of the Hamiltonians $H_{n,t}$ is determined by the representation theory of the Clifford group. With little additional work, we obtain the following lemma about the ground space structure of our Hamiltonians.

#### Lemma 8

(Lower bound to spectral gap). Let the Hamiltonian $H_{n,t}$ be as in Eq. (72) and assume that $n\ge 12t$. Then, $H_{n,t}$ has a spectral gap satisfying

75 $$\begin{aligned} \Delta (H_{n,t})\ge \frac{\Delta (H_{12t,t})}{48t}. \end{aligned}$$

#### Proof

We make use of the Nachtergaele lemma. We have to verify the three conditions of Lemma 14. As already stated in Ref. [61], the first two conditions hold directly for translation-invariant local Hamiltonians as in our case.

1. The first condition immediately follows from the fact that we consider a translation-invariant 2-local Hamiltonian. It is fulfilled for any choice of $l\ge 2$ and $d_l=l-1$.
2. The second condition follows again for all $l\ge 2$ and the choice $Q_l=l$, since $H_{[q-l+1,q]}$ is a sum of positive semi-definite operators for all $q\ge l$ with spectrum that does not depend on *q* due to translation-invariance. Thus, we can set
  139 $$\begin{aligned} \gamma _l:= \Delta (H_{[q-l+1,q]}) > 0. \end{aligned}$$
3. The third condition requires a calculation and a non-trivial choice of *l*. We have to bound the quantity
  140 $$\begin{aligned} R_{q,l} := \left\| G_{[q-l+2,q+1]}\left( G_{[1,q]}-G_{[1,q+1]}\right) \right\| _{\infty }, \end{aligned}$$
  for all $q\ge Q_l=l$. Here, $G_{[p,q]}$ denotes the orthogonal projector onto the ground space of $H_{[p,q]}$. Note that this ground space is simply a suitable translation of the Clifford commutant ${{\,\textrm{Cl}\,}}(k)'$ for $k=q-p+1$ as shown in Lemma 7. Recall that it comes with a non-orthogonal basis $Q_T^{\otimes k}$, where
  141 $$\begin{aligned} Q_T := \frac{r(T)}{\Vert r(T)\Vert _2} = 2^{-t/2} r(T), \quad T \in \Sigma _{t,t}. \end{aligned}$$
  Moreover, the projector $G_[p,q]$ is also simply a translation of the Clifford projector $P_{{{\,\textrm{Cl}\,}}(k)}$ projecting onto ${{\,\textrm{Cl}\,}}(k)'$. From the discussion in Sect. 6.1, we know that the Clifford frame operator
  142 $$\begin{aligned} S_{{{\,\textrm{Cl}\,}}(k)}:= \sum _T \left. \left| {Q_T}\right. \right) \!\! \left. \left( {Q_T}\right. \right| ^{\otimes k}, \end{aligned}$$
  is a suitable approximation to $P_{{{\,\textrm{Cl}\,}}(k)}$ when *k* is large enough. Concretely, we have by Lem. 12:
  143 $$\begin{aligned} \left\| S_{{{\,\textrm{Cl}\,}}(k)}-P_{{{\,\textrm{Cl}\,}}(k)} \right\| _\infty \le (-2^{-k};2)_{t-1} - 1. \end{aligned}$$
  Defining the shorthand notation $s_t(k)=(-2^{-k};2)_{t-1}$, we in particular get the bound
  144 $$\begin{aligned} \left\| S_{{{\,\textrm{Cl}\,}}(k)} \right\| _\infty \le \left\| S_{{{\,\textrm{Cl}\,}}(k)}-P_{{{\,\textrm{Cl}\,}}(k)} \right\| _\infty + \left\| S_{{{\,\textrm{Cl}\,}}(k)} \right\| _\infty \le s_t(k), \end{aligned}$$
  Let us introduce the shorthand notation $G_q:=G_{[1,q]}\equiv P_{{{\,\textrm{Cl}\,}}(q)}$, $S_q = S_{[1,q]}\equiv S_{{{\,\textrm{Cl}\,}}(q)}$, and $G_{q,l}:=G_{[q-l+2,q+1]}$, $S_{q,l}:=S_{[q-l+2,q+1]}$ for translations of the Clifford projector and frame operator, respectively. Notice that $G_q-G_{q+1}$ is an orthogonal projector as the support of $G_{q+1}$ is by definition contained in that of $G_q$. Therefore, restricted to the support of $G_q$, the operator $G_q-G_{q+1}$ projects onto the orthogonal complement of the support of $G_{q+1}$. Combining this fact with the above inequalities, we find
  145 $$\begin{aligned} R_{q,l}&= \left\| G_{q,l}\left( G_{q}-G_{q+1}\right) \right\| _\infty \nonumber \\&\le \left\| (G_{q,l}-S_{q,l})(G_q-G_{q+1}) \right\| _\infty + \left\| S_{q,l}(G_q-G_{q+1}) \right\| _\infty \nonumber \\&\le s_t(l) - 1 + \left\| S_{q,l}(S_q-S_{q+1}) \right\| _\infty + \left\| S_{q,l}(G_q-S_q) \right\| _\infty \nonumber \\&\quad + \left\| S_{q,l}(G_{q+1}-S_{q+1}) \right\| _\infty \nonumber \\&\le \left\| S_{q,l}(S_q-S_{q+1}) \right\| _\infty + s_t(l) - 1 + s_t(l)\left( s_t(q) + s_t(q+1) -2 \right) \nonumber \\&{\mathop {\le }\limits ^{q\ge l}} \left\| S_{q,l}(S_q-S_{q+1}) \right\| _\infty + \left( s_t(l) - 1 \right) \left( 2 s_t(l) + 1 \right) \nonumber \\&= \left\| \sum _{T\in \Sigma _{t,t}} \left. \left| {Q_T}\right. \right) \!\! \left. \left( {Q_T}\right. \right| ^{\otimes (q-l+1)} \otimes Y_T \right\| _\infty + \left( s_t(l) - 1 \right) \left( 2 s_t(l) + 1 \right) , \end{aligned}$$
  where the operator $Y_T$ can be straightforwardly computed as
  146 $$\begin{aligned} Y_T&:= \sum _{T'\ne T}\left( \left( Q_{T'} \big | Q_{T} \right) ^{l-1} \left. \left| {Q_{T'}}\right. \right) \!\! \left. \left( {Q_{T}}\right. \right| ^{\otimes (l-1)}\right) \otimes \nonumber \\&\qquad \Big ( \left. \left| {Q_{T'}}\right. \right) \!\! \left. \left( {Q_{T'}}\right. \right| \big (\textrm{id}-\left. \left| {Q_{T}}\right. \right) \!\! \left. \left( {Q_{T}}\right. \right| \big ) \Big ). \end{aligned}$$
  Invoking the synthesis operators
  147 $$\begin{aligned} V_{k} = \sum _T \left. \left| {Q_T^{\otimes k}}\right. \right) \!\! \left. \left\langle {e_T}\right. \right| :\; {\mathbb {C}}^{|\Sigma _{t,t}|}\longrightarrow {{\,\textrm{Cl}\,}}(k)', \end{aligned}$$
  introduced in Lemma 12, one can bound the above norm as
  148 $$\begin{aligned} \left\| \sum _{T} \left. \left| {Q_T}\right. \right) \!\! \left. \left( {Q_T}\right. \right| ^{\otimes (q-l+1)} \otimes Y_T \right\| _\infty&= \left\| \sum _{T} V_{q-l+1}\left. \left| {e_T}\right. \right\rangle \!\! \left. \left\langle {e_T}\right. \right| V_{q-l+1}^\dagger \otimes Y_T \right\| _\infty \nonumber \\&\le \left\| V_{q-l+1}V^\dagger _{q-l+1} \right\| _\infty \left\| \sum _{T} \left. \left| {e_T}\right. \right\rangle \!\! \left. \left\langle {e_T}\right. \right| \otimes Y_T \right\| _\infty \nonumber \\&= \left\| S_{q-l+1} \right\| _\infty \max _T \left\| Y_T \right\| _\infty \nonumber \\&\le s_t(q-l+1) \left( s_t(l-1) - 1 \right) . \end{aligned}$$
  Thus, we arrive at
  149 $$\begin{aligned} R_{q,l}&\le s_t(q-l+1) \left( s_t(l-1) - 1 \right) + \left( s_t(l) - 1 \right) \left( 2 s_t(l) + 1 \right) \nonumber \\&\le s_t(1) \left( s_t(l-1) - 1 \right) + \left( s_t(l) - 1 \right) \left( 2 s_t(l) + 1 \right) . \end{aligned}$$
  For $l+1\ge t+\log _2(t)$, we can use Lemma 11 to get:
  150 $$\begin{aligned} R_{q,l}&\le t 2^{t-l+1} \left( 1 + t 2^{t-1} \right) + t 2^{t-l} \left( 3 + t 2^{t-l}\right) \nonumber \\&= t^2 2^{2t-l}\left( \frac{5}{t} 2^{-t} + 2^{-l} + 1 \right) \nonumber \\&\le 4 t^2 2^{2t-l}. \end{aligned}$$
  Finally choose any $l \ge 4t + 4\log _2(t) + 6$, then we find
  151 $$\begin{aligned} l \le \frac{4^{l-2t}}{64t^2} \quad \Rightarrow \quad R_{q,l} \le 4 t^2 2^{2t-l} \le \frac{1}{2\sqrt{l}} < \frac{1}{\sqrt{l}}, \quad \forall q \ge l. \end{aligned}$$
  In particular, we can choose $l = 12t$, $\varepsilon _l = 1/2\sqrt{l}$ to get the desired bound in Lemma 14$\forall q \ge l$.

Hence, for the choices $l = 12t$, $d_l=l-1$, $Q_l=l$, $\gamma _l = \Delta (H_{12t,t})$ and $\varepsilon _l = 1/2\sqrt{l}$, Lemma 14 gives the claimed bound on the spectral gap:

152 $$\begin{aligned} \Delta (H_{n,t}) \ge \frac{\gamma _l}{d_l}\left( 1-\varepsilon _l^2 \sqrt{l}\right) \ge \frac{\Delta (H_{12t,t})}{48t}. \end{aligned}$$

$$\square $$

## Summary and Open Questions

We have found that a number of non-Clifford gates independent of the system size suffices to generate $\varepsilon$-approximate unitary *t*-designs. This is surprising, conceptually interesting and practically relevant: After all, it is the main objective in quantum gate synthesis to minimize the number of non-Clifford gates in a circuit implementation of a given unitary. There are multiple open questions and ways to continue this work:

- Similar to the result in Ref. [24], the scaling in *n* is near to optimal, the scaling in *t* can probably be improved.
- Another natural open question is whether the condition $n=O(t^2)$ can be lifted. Notably, this is reminiscent to the situation discussed in Ref. [69], where the improved scaling can be proven only in the regime $t=o(n^{\frac{1}{2}})$. In this work, the condition $n=O(t^2)$ is related to the approximate orthogonality of the Lagrangian subspace. We use this fact repeatedly and in different flavours, but we can only prove it in this regime. In fact, in Lemma 12 we use the same technique that has been used in Ref. [24] to prove approximate orthogonality of permutations in the regimes $t\le 2^{O(0.4n)}$. However, the commutant of the Clifford group is far larger than the span of permutations and we suspect that this bound is tight. Nevertheless, we cannot rule out that similar results can be proven without exploiting approximate orthogonality. This likely requires a detailed understanding of the representation theory of the Clifford group.
- Our result holds for additive errors in the diamond norm. For relative errors, our bounds can be used to obtain a quadratic advantage in the number of non-Clifford gates in Corollary 1. This still allows the density of non-Clifford gates to go to zero in the thermodynamic limit, but is not system-size independent anymore. In fact, it has been proven in Ref. [70] that this scaling is optimal for relative errors. It would be interesting to delineate more precisely for which notions of approximations a system-size independent result holds.
- We strongly expect that the results can be generalized to qu*d*its for arbitrary *d*, giving rise to analogous conclusions concerning an independence of the system size for additive errors in the diamond norm.

We hope the present work stimulates such endeavors.

## Appendix A: Unitary *t*-designs

In the following, we review the concept of a *unitary* *t*-*design* [5–7], giving different but equivalent definitions which prove to be useful in different contexts. They also serve as starting point to explore connections to other mathematical fields, e. g. representation theory. To this end, let us introduce some notation. Define $\mu _\textrm{H}$ to be the (normalized) Haar measure on ${{\,\textrm{U}\,}}(d)$ and let ${{\,\textrm{Hom}\,}}_{(t,t)}({{\,\textrm{U}\,}}(d))$ be the space of homogeneous polynomials of degree *t* in both the entries of $U\in {{\,\textrm{U}\,}}(d)$ as well as ${\overline{U}}$.

### Definition 10

(*Unitary* *t*-*design*). A probability measure $\nu$ on ${{\,\textrm{U}\,}}(d)$ is called a *unitary* *t*-*design* if the following holds for all $p\in {{\,\textrm{Hom}\,}}_{(t,t)}({{\,\textrm{U}\,}}(d))$:

A1 $$\begin{aligned} \int _{{{\,\textrm{U}\,}}(d)} p(U)\,\textrm{d}\nu (U) = \int _{{{\,\textrm{U}\,}}(d)} p(U)\,\textrm{d}\mu _\textrm{H}(U). \end{aligned}$$

A subset $D\subseteq {{\,\textrm{U}\,}}(d)$ is called a *unitary* *t*-*design*, if it comes with a probability measure $\nu _D$ which, continued trivially to ${{\,\textrm{U}\,}}(d)$, is a unitary *t*-design. In particular, if *D* is finite, $\nu _D$ is usually taken to be the (normalized) counting measure.

It might not come as a surprise that Def. 10 has not to be checked for any polynomial. Since any homogeneous polynomial $p\in {{\,\textrm{Hom}\,}}_{(t,t)}({{\,\textrm{U}\,}}(d))$ can be linearized as

A2 $$\begin{aligned} p(U) = {{\,\textrm{Tr}\,}}\left( A U^{\otimes t,t}\right) , \qquad U^{\otimes t,t}:=U^{\otimes t}\otimes {\overline{U}}^{\otimes t}, \end{aligned}$$

the defining Eq. (A1) becomes

A3 $$\begin{aligned} M_t(\nu ):=\int _{{{\,\textrm{U}\,}}(d)} U^{\otimes t,t}\,\textrm{d}\nu (U) = \int _{{{\,\textrm{U}\,}}(d)} U^{\otimes t,t}\,\textrm{d}\mu _\textrm{H}(U) =: M_t(\mu _\textrm{H}). \end{aligned}$$

Thus $\nu$ is a unitary *t*-design if and only if its moment operator $M_t(\nu )$ agrees with the one of the Haar measure. Note that the operators $U^{\otimes t,t}$ are the matrix representation of the *t*-diagonal adjoint action ${{\,\textrm{Ad}\,}}(U^{\otimes t}) = U^{\otimes t}\bullet (U^\dagger )^{\otimes t}$ with respect to the standard basis $\left. \left| {i}\right. \right\rangle \!\! \left. \left\langle {j}\right. \right|$ of $L({\mathbb {C}}^d)$. Thus, this can be equivalently stated as equality of the twirls $\textrm{M}_t(\nu )=\textrm{M}_t(\mu _\textrm{H})$ over the two measures.

A particularly fruitful theory of designs is possible in the case where the design $(G,\nu )$ itself constitutes a (locally compact) subgroup $G\subseteq {{\,\textrm{U}\,}}(d)$ and $\nu$ is the normalized Haar measure on *G*. Following Ref. [38], we call these *unitary* *t*-*groups*. In this case, we see that Eq. (A3) implies that the trivial isotype of the representation $G \ni g \mapsto {{\,\textrm{Ad}\,}}_g^{\otimes t}$ shall agree with the trivial isotype of ${{\,\textrm{U}\,}}(d) \ni U \mapsto {{\,\textrm{Ad}\,}}_U^{\otimes t}$. Since the trivial isotype exactly corresponds to the commutant of the respective diagonal representations $\tau _t: U \mapsto U^{\otimes t}$, this is equivalent to the statement that the commutant of the representation $\tau _t$ agrees with the commutant of the restriction $\tau _t|_G$. However, this is the case if and only if $\tau _t|_G$ decomposes into the same irreducible representations as $\tau _t$.

## Appendix B: Representations of the Unitary Group

The representation theory of the unitary group can be understood using the theory of highest weight for compact Lie groups, see, for example Refs. [71–73]. We present a short summary of the part relevant to us here. Let $\rho$ be an irreducible representation of ${{\,\textrm{U}\,}}(d)$, and consider the restriction $\rho |_{D(d)}$ to the diagonal subgroup $D(d)\simeq (S^1)^{\times d}$ (which is a so-called *maximal torus* in ${{\,\textrm{U}\,}}(d)$). In general, this is a reducible representation of *D*(*d*). Since *D*(*d*) is Abelian, $\rho |_{D(d)}$ decomposes into one-dimensional irreducible representations , i. e. characters of $D(d)\simeq (S^1)^{\times d}$. Those are of the form $\chi _u(\theta ) := e^{i u^T \theta }$ for some vector $u\in {\mathbb {Z}}^d$, and thus we find

B1 $$\begin{aligned} \rho |_{D(d)} \simeq \bigoplus _{u\in {\mathbb {Z}}^d} \chi _u\otimes \mathbbm {1}_{m_u}, \end{aligned}$$

where $m_u \in {\mathbb {N}}$ are multiplicities. The vectors *u* for which $m_u\ne 0$ are called the *weights* of $\rho$. Introducing a lexicographical ordering of the weights, we call a weight *u* higher than the weight *v* if $u>v$. The *theorem of the highest weight* states that any irreducible representation $\rho$ has a highest weight and that irreducible representations with the same highest weight are isomorphic. Thus, irreducible representations are unambiguously labeled by their highest weight. Next, let us consider the tensor product $\pi _u\otimes \pi _v$ of two irreducible representations labeled by their highest weights *u* and *v*. One can easily check that the weights of irreducible representations in $\pi _u\otimes \pi _v$ have to be sums of weights of $\pi _u$ and $\pi _v$. In particular, the highest weight of all irreducible representations is at most $u+v$.

As a relevant example consider the (irreducible) defining representation $\rho : U \mapsto U$ of *U*(2). Its restriction to the diagonal subgroup $S^1\times S^1$ decomposes as

$$\begin{aligned} \rho |_{S^1\times S^1} \simeq \chi _{e_1}\oplus \chi _{e_2}, \end{aligned}$$

with highest weight $e_1=(1,0)$. Using ${\bar{\chi }}_u = \chi _{-u}$, the highest weight of the complex conjugate representation ${\bar{\rho }}:\,U\mapsto {\bar{U}}$ can be immediately determined as $(0,-1)$. Hence, the weights of $\rho \otimes {{\bar{\rho }}}$ are $\{(0,0), (1,-1), (-1,1)\}$. Here, (0, 0) is the highest weight of the trivial irreducible representation and $(1,-1)$ the highest weight of the adjoint irrep. Finally, all irreducible representations appearing in $(\rho \otimes {{\bar{\rho }}})^{\otimes t}$ have weights *w* satisfying $(-t,t)\le w\le (t,-t)$ and, in particular,

$$\begin{aligned} w = \sum _{i=1}^tu_i \end{aligned}$$

where $u_i \in \{(0,0), (1,-1), (-1,1)\}$. It follows that the Euclidean norm of these weights is at most $\sqrt{2}t$.

## Appendix C: Converse Bounds for Estimates in Sect. 6.3

Here, we collect various tightness results that limit the degree by which the estimates in Sect. 6.3 can be improved. The bound in Proposition 4 is tight in many cases. Most interestingly, the anti-identity [42]

C1 $$\begin{aligned} {{\overline{\mathbbm {1}}}}= \begin{pmatrix} 0 &{} 1 &{} \cdots &{} 1\\ 1 &{} \ddots &{} \ddots &{} \vdots \\ \vdots &{} \ddots &{} \ddots &{} 1\\ 1 &{} \cdots &{} 1 &{} 0 \end{pmatrix} \in O_t\, , \end{aligned}$$

meets the bound if both

C2 $$\begin{aligned} r=t-1 \qquad \text {and}\qquad t/2=(r+1)/2 \qquad \text { are odd}. \end{aligned}$$

Indeed, the anti-identity flips the components of the input if its parity is odd, and leaves the input invariant if the parity is even. The flipping step preserves the Hamming weight if and only if $h(a)=t/2$. Thus

$$\begin{aligned} \textrm{Pr}[ h(Oa) = h(a) ]&= \textrm{Pr}[ h(a) \text { even}] + \textrm{Pr}[ h(a) \text { odd} \wedge h(a)=t/2] \nonumber \\&= \textrm{Pr}[ h(a) \text { even}] + \textrm{Pr}[h(a)=t/2]{} & {} \text {(using (C2))} \nonumber \\&= \frac{1}{2} + 2^{-t} {{t}\atopwithdelims (){t/2}}\nonumber \\&= \frac{1}{2} + 2^{-(r+1)} {{r+1}\atopwithdelims (){(r+1)/2}}. \end{aligned}$$

Likewise, both estimates in Proposition 5 are tight. The first bound is saturated for $N=\{0, (1,1,1,1)\}$. Indeed, $N^\perp$ is the space of all even-weight elements of ${\mathbb {F}}_2^4$. The only non-trivial element of *N* is (1, 1, 1, 1) and adding it to an even-weight vector changes its weight if and only if the vector is in *N* itself. But $|N|/|N^\perp |=1/4$. In an exactly analogous way, the second bound is tight for the stochastic Lagrangian with left and right defect spaces equal to the same *N*. As detailed in Example 4.27 of Ref. [42], this stochastic Lagrangian is the one identified in Ref. [74] as the sole non-trivial one in case of $t=4$.

In contrast, we do not know (but suspect) that we pay a price by restricting from the full Haar symmetrizer to the one over diagonal matrices in Eq. (123). For the two cases that saturate the bounds in Proposition 4 and Proposition 5, we can compute the full projection explictily and show that at least there, Eq. (123) indeed fails to be tight.

One can expand the anti-id ${{\overline{\mathbbm {1}}}}$ in terms of Pauli operators [42]

C3 $$\begin{aligned} {{\overline{\mathbbm {1}}}} = \frac{1}{2} \big (\mathbbm {1}^{\otimes t} + X^{\otimes t} + Y^{\otimes t} + Z^{\otimes t}\big ). \end{aligned}$$

Then

C4 $$\begin{aligned} 2^{-t} \big (r({{\overline{\mathbbm {1}}}}), P_H[r({{\overline{\mathbbm {1}}}})]\big )&= 2^{-t} \int {{\,\textrm{Tr}\,}}r({{\overline{\mathbbm {1}}}})U^{\otimes t} r({{\overline{\mathbbm {1}}}})^\dagger (U^\dagger )^{\otimes t}\; \textrm{d} {\mu _H(U)} \nonumber \\&= 2^{-t-2} \sum _{i,j=0}^3 \int {{\,\textrm{Tr}\,}}\sigma _i^{\otimes t} U^{\otimes t} \sigma _j^{\otimes t} (U^\dagger )^{\otimes t} \, \textrm{d} {\mu _H(U)} \nonumber \\&= 2^{-t-2} \sum _{i,j} \int \Big ({{\,\textrm{Tr}\,}}\sigma _i U \sigma _j U^\dagger \Big )^{t} \, \textrm{d} {\mu _H(U)}\nonumber \\&= 2^{-2} + 2^{-t-2} \sum _{i,j\ne 0} \int \Big ({{\,\textrm{Tr}\,}}\sigma _i U \sigma _j U^\dagger \Big )^{t} \, \textrm{d} {\mu _H(U)}\nonumber \\&= 2^{-2} + 2^{-2} 9 \frac{1}{4\pi } \int _{S^2} x_1^{t} \textrm{d} x \nonumber \\&= \frac{1}{4} + \frac{9}{4} \frac{1}{4\pi } \frac{4\pi }{1+t} = \frac{1}{4}\Big (1+\frac{9}{t+1}\Big ), \end{aligned}$$

where in (C4), we have interpreted the Haar integral over inner products of Paulis as an integral over the Bloch sphere and in the next line, used the formula from [75]. For $t=2$, Eq. (C1) is just the swap operator (i.e., a permutation), and the formula gives 1, as it should. The smallest non-trivial case is $t=6$ [42] , where we get roughly $0.571<0.65$.

Next, we consider the CSS code $P_N$ for $N=(1,1,1,1)$. We use the results in Sect. 3 of Ref. [74]. For a given partition $\lambda$, let $W_\lambda$ be the associated Weyl module and $S_\lambda$ the Schur module. As in Ref. [74], let $W^+_\lambda \subset W_\lambda$ be the subspace such that

$$\begin{aligned} \big (W_\lambda \otimes S_\lambda \big )\cap \textrm{range}\, P_N = W_\lambda ^+ \otimes S_\lambda . \end{aligned}$$

For the projection operators onto the various spaces, we write $P_\lambda$ (Schur module), $Q_\lambda$ (Weyl module), and $Q^+_\lambda$ (the subspace defined above). Then [74]

$$\begin{aligned} P_N = \sum _\lambda Q^+_\lambda \otimes P_\lambda . \end{aligned}$$

By Schur’s Lemma,

$$\begin{aligned} P_H[P_N] = \sum _\lambda c_\lambda Q_\lambda \otimes P_\lambda , \end{aligned}$$

for suitable coefficients $c_\lambda$, which are seen to equal $c_\lambda = D^+_\lambda /D_\lambda$ by the fact that Haar averaging preserves the trace. Hence, using Table 1 of Ref. [74] for $d=2$,

$$\begin{aligned} 2^{-t+2\dim N} (P_N, P_\textrm{H}[P_N]) = 2^{-2} \sum _\lambda \frac{d_\lambda (D^+_\lambda )^2 }{D_\lambda } = \frac{7}{10} < \frac{7}{8}. \end{aligned}$$

## Appendix D: Saturation of Higher Rényi-entropies in *K*-interleaved Clifford Circuits

Consider the Rényi-entropies which are defined as

D1 $$\begin{aligned} S_{\alpha }(\rho ):=\frac{1}{1-\alpha }\log \textrm{Tr}[\rho ^{\alpha }] \end{aligned}$$

for $\alpha >0$. For $\alpha \searrow 1$ the standard von Neumann entropy is recovered. Here, we are interested in the entanglement properties of random state vectors $|\psi \rangle$ on *n* qubits. We consider a bi-partition of the *n* qubits into a set *A* consisting of constantly many qubits $n_A$ and a set *B* of $n_B=n-n_A$ many qubits that constitutes the complement of *A*. To derive concentration bounds on these quantities over random ensembles of states, we study the “higher purities” $\textrm{Tr}[\rho ^{\alpha }]$ for positive integer $\alpha$ in more detail. First, we compute the Haar average of this quantity. Let $\pi _{\textrm{cyc}}\in S_{\alpha }$ be any full $\alpha$-cycle. We compute

D2 $$\begin{aligned} \begin{aligned} {\mathbb {E}}_{U\sim \mu _H}\textrm{Tr}[\rho _A^{\alpha }]&= {\mathbb {E}}_{U\sim \mu _H}\textrm{Tr}\left[ \textrm{Tr}_B[|\psi \rangle \langle \psi |]^{\alpha }\right] \\&={\mathbb {E}}_{U\sim \mu _H}\textrm{Tr}\left[ r(\pi _{\textrm{cyc}})_A\otimes \mathbbm {1}_B(|\psi \rangle \langle \psi |)^{\otimes \alpha }\right] \\&= {2^n+\alpha -1\atopwithdelims ()\alpha }^{-1}\textrm{Tr} \left[ r(\pi _{\textrm{cyc}})_A\otimes \mathbbm {1}_B P_{\textrm{sym},\alpha }\right] \\&={2^n+\alpha -1\atopwithdelims ()\alpha }^{-1}\alpha !^{-1}\sum _{\sigma \in S_{\alpha }}\textrm{Tr}\left[ r(\pi _{\textrm{cyc}}\circ \sigma )_A\otimes r(\sigma )_B\right] \\&={2^n+\alpha -1\atopwithdelims ()\alpha }^{-1}\alpha !^{-1}\sum _{\sigma \in S_{\alpha }} 2^{n_A \#\textrm{cyc}(\pi _{\textrm{cyc}}\circ \sigma )}2^{n_B \#\textrm{cyc}(\sigma )}\\&=\frac{1}{2^n(2^n+1)\ldots (2^n+\alpha -1)}\sum _{\sigma \in S_{\alpha }} 2^{n_A \#\textrm{cyc}(\pi _{\textrm{cyc}}\circ \sigma )}2^{n_B \#\textrm{cyc}(\sigma )}\\&=\frac{2^{\alpha n_B}2^{n_A}}{2^n(2^n+1)\ldots (2^n+\alpha -1)}+O(2^{-n_B})\\&=2^{-(\alpha -1)n_A}+O(2^{-n_B}), \end{aligned} \end{aligned}$$

where $O(2^{-n_B})$ depends on $\alpha$. Therefore, up to an exponentially small correction, the average higher purity is minimal.

Next, we compute the same average over an additive $\varepsilon$-approximate unitary *t*-design. Recall that this is a probability distribution $\nu$ such that

D3 $$\begin{aligned} ||M_t(\nu )-M_t(\mu _H)||_{\Diamond }\le \varepsilon . \end{aligned}$$

By definition of the diamond norm, this also implies

D4 $$\begin{aligned} ||M_t(\nu )-M_t(\mu _H)||_{1\rightarrow 1}\le \varepsilon . \end{aligned}$$

From this, we obtain

D5 $$\begin{aligned} {\mathbb {E}}_{U\sim \nu }\textrm{Tr}[\rho _A^{\alpha }]&= {\mathbb {E}}_{U\sim \nu }\textrm{Tr}\left[ \textrm{Tr}_B[|\psi \rangle \langle \psi |]^{\alpha }\right] \nonumber \\&=\textrm{Tr}\left[ r(\pi _{\textrm{cyc}})_A\otimes \mathbbm {1}_B{\mathbb {E}}_{U\sim \nu }(|\psi \rangle \langle \psi |)^{\otimes \alpha }\right] \nonumber \\&\le \textrm{Tr}\left[ r(\pi _{\textrm{cyc}})_A\otimes \mathbbm {1}_B{\mathbb {E}}_{U\sim \mu _H}(|\psi \rangle \langle \psi |)^{\otimes \alpha }\right] \nonumber \\&\quad +\left| \textrm{Tr}[r(\pi _{\textrm{cyc}})_A\otimes \mathbbm {1}_B(M_t(\nu )-M_t(\mu _H))\left[ (|\psi _0\rangle \langle \psi _0|)^{\otimes \alpha }\right] \right| \nonumber \\&\le \textrm{Tr}\left[ r(\pi _{\textrm{cyc}})_A\otimes \mathbbm {1}_B{\mathbb {E}}_{U\sim \mu _H}(|\psi \rangle \langle \psi |)^{\otimes \alpha }\right] \nonumber \\&\quad +\left| \left| (M_t(\nu )-M_t(\mu _H))\left[ (|\psi \rangle \langle \psi |)^{\otimes \alpha }\right] \right| \right| _1\nonumber \\&\le 2^{-(\alpha -1)n_A}+O(2^{-n})+\varepsilon . \end{aligned}$$

It suffices to insert $C(K)\log ^2(t)(t^4+t\log (1/\varepsilon ))$ non-Clifford gates into random Clifford circuits to generate an additive $\varepsilon$-approximate *t*-designs. Therefore, we can choose $\varepsilon =2^{-2(\alpha -1)n_A}$ and $t=\alpha$ and find that a *K*-interleaved Clifford circuit with $k=C(K)\log ^2(\alpha )(\alpha ^4+2(\alpha -1)n_A)$ satisfies

D6 $$\begin{aligned} {\mathbb {E}}_{U\sim \sigma ^{*k}}\textrm{Tr}[\rho _A^{\alpha }]\le & {} (1-2^{-(\alpha -1)n_A})2^{-(\alpha -1)n_A}+O(2^{-n})\nonumber \\\le & {} (1-2^{-(\alpha -1)n_A}-O(2^{-n}))2^{-(\alpha -1)n_A}. \end{aligned}$$

Therefore, for every constant $n_A$ and $\alpha$, there is a classically simulable ensemble of quantum circuits that generate essentially minimal higher purities on average.

## Footnotes

1 We use the set $\{K, K^\dagger , \mathbbm {1}\}$ instead of just $\{K\}$ for technical reasons: Making the set closed under the adjoint causes the moment operator to be Hermitian. The identity is included to ensure that the concatenation of two random elements has a non-vanishing probability of producing a non-Clifford gate—a property that will slightly simplify the proof. Of course, in a physical realization, identity gates and the following Clifford operation are redundant and need not be implemented.

## Declarations

### Data Availability Statement

No data was generated in this work.
